# Using high-amplitude and focused transcranial alternating current stimulation to entrain physiological tremor

**DOI:** 10.1038/s41598-018-23290-w

**Published:** 2018-03-21

**Authors:** Ahmad Khatoun, Jolien Breukers, Sara Op de Beeck, Ioana Gabriela Nica, Jean-Marie Aerts, Laura Seynaeve, Tom Haeck, Boateng Asamoah, Myles Mc Laughlin

**Affiliations:** 1 ExpORL, Department of Neurosciences, KU Leuven, B-3000 Leuven, Belgium; 2 Division Animal and Human Health Engineering, Department of Biosystems, Faculty of Bioscience Engineering, KU Leuven, B-3000 Leuven, Belgium; 3 Department of Neurology, Laboratory for Epilepsy Research, University Hospitals & KU Leuven, B-3000 Leuven, Belgium; 4 Medical Imaging Research Center (MIRC), KU Leuven, B-3000 Leuven, Belgium; 5 Center for Processing Speech and Images (PSI), Department of Electrical Engineering (ESAT), KU Leuven, B-3000 Leuven, Belgium

## Abstract

Transcranial alternating current stimulation (tACS) is a noninvasive neuromodulation method that can entrain physiological tremor in healthy volunteers. We conducted two experiments to investigate the effectiveness of high-amplitude and focused tACS montages at entraining physiological tremor. Experiment 1 used saline-soaked sponge electrodes with an extra-cephalic return electrode and compared the effects of a motor (MC) and prefrontal cortex (PFC) electrode location. Average peak-amplitude was 1.925 mA. Experiment 2 used gel-filled cup-electrodes in a 4 × 1 focused montage and compared the effects of MC and occipital cortex (OC) tACS. Average peak-amplitude was 4.45 mA. Experiment 1 showed that unfocused MC and PFC tACS both produced phosphenes and significant phase entrainment. Experiment 2 showed that focused MC and OC tACS produced no phosphenes but only focused MC tACS caused significant phase entrainment. At the group level, tACS did not have a significant effect on tremor amplitude. However, with focused tACS there was a significant correlation between phase entrainment and tremor amplitude modulation: subjects with higher phase entrainment showed more tremor amplitude modulation. We conclude that: (1) focused montages allow for high-amplitude tACS without phosphenes and (2) high amplitude focused tACS can entrain physiological tremor.

## Introduction

Transcranial alternating current stimulation (tACS) is a noninvasive neuromodulation method in which current is passed through scalp electrodes to create a weak (<1 V/m), sinusoidally varying electric field in the cortex^1,2^. Work in brain slices shows that similarly weak electric fields polarize the membrane potential in cortical neurons^3^, while animal studies show that these fields can entrain firing patterns in cortical neurons^4^. When applied to healthy volunteers, tACS modulates brain oscillations^5,6^ which are believed to then modulate perception^7^, cognition^8^, and motor function^9,10^. The mechanisms of membrane polarization, followed by spike entrainment are assumed to underpin the human behavioral effects.

An interesting application of tACS is tremor control. Two studies have shown that tACS can entrain physiological tremor in healthy volunteers^11,12^, but found no effect on tremor amplitude. A ground breaking study in patients showed that tACS reduces Parkinsonian tremor amplitude by around 50%^13^. All three studies measured tremor via an accelerometer and applied tACS at the tremor frequency.

tACS studies generally use peak-amplitudes below 1 mA (i.e. 2 mA peak-to-peak). However, we know from brain slice and *in-vivo* work that increasing the electric field strength increases membrane polarization and spike entrainment^3,4^. Thus, applying higher amplitude tACS in humans will create a stronger electric field in the brain, which should cause stronger membrane polarization and spike entrainment, potentially leading to an increased behavioral effect. In line with this, a recent meta-analysis indicated stronger behavioral effects were associated with higher tACS amplitudes^14^. However, the feasibility of applying tACS at peak-amplitudes above 1 mA has not been thoroughly studied.

An assumption in human tACS studies is that the behavioral effects are caused by modulation of cortical neurons under the electrode. However, a potential confound is that low-frequency tACS (<40 Hz) can cause phosphenes^15^. The tACS electric field is broad and spreads out across the head. Evidence suggests that field spread to the retina (which is not insulated by the scalp and skull) causes phosphenes^16^ and it is known that tremor entrains to flickering light^11^. Interestingly, a recent study demonstrated that a focused concentric ring montage can limit field spread and reduce phosphenes^10^.

The aim of this study was to investigate the feasibility of using high-amplitude tACS to entrain physiological tremor in healthy volunteers and to assess the effect of different electrode montages and stimulation sites. In-house safety testing was done before performing these experiments (see Supplementary Methods). Two experiments were conducted: Experiment 1 employed an unfocused montage with conventional saline-soaked sponge electrodes to compare motor cortex (MC) tACS to prefrontal cortex (PFC) tACS. The maximum peak-amplitude was 2 mA. We hypothesized that unfocused MC tACS would have stronger tremor entrainment but less intense phosphenes than unfocused PFC tACS. Experiment 2 employed a focused 4 × 1 montage with gel-filled cup-electrodes to compare MC tACS to occipital cortex (OC) tACS. The maximum peak-amplitude was 5 mA. We hypothesized that focused MC tACS would cause tremor entrainment while OC tACS would not; and that no phosphenes would be observed with either of the focused montages. In both experiments, five outcome measurements were assessed: phosphenes threshold and intensity; physiological tremor phase entrainment and amplitude modulation; number of healthy volunteers showing an increase in phase entrainment. Additionally, an electro-anatomical computational model was used to estimate cortical electric field and compare the electric field distributions from the two montages.

## Materials and Methods

### Experiment 1

#### Subjects

Ten healthy volunteers participated in the Experiment 1 (4 females; age 26 ± 6). All were right-handed except for one (self-reported). The experiment was approved by the Medical Ethics Committee UZ KU Leuven/Research (S57869) and was performed in accordance with the relevant guidelines and regulations (clinicaltrials.gov registration number NCT03337334, date 08/11/2017). Informed consent to participate in the experiment was obtained from all healthy volunteers.

#### tACS procedure

Experiment 1 compared the effects of MC tACS to PFC tACS. Three square saline-soaked sponge electrodes (Rogue Resolutions, UK) were used: one 5 × 5 cm electrode was placed over each stimulation site: MC and PFC. One 10 × 10 cm return electrode was placed around the ankle, contralateral to the targeted MC. For MC the small electrode was centered between C1 (10–20 system nomenclature) and C3 (C2 and C4 for left-handed) and for PFC the small electrode was contralateral to the MC electrode (F5/F6). Electrodes were connected to a DS5 current source stimulator (Digitimer, Hertfordshire, UK); driven by a voltage waveform generated on a data acquisition card (1024 Hz, NI USB-6216, National Instruments, Austin, TX) and controlled via custom written MATLAB R2014a software (Mathworks, Natwick, MA). Our in house safety review (see Supplementary Information) indicated that tACS at these amplitudes and time durations should be safe – it will not cause skin redness, irritation or burns. The risks are essentially similar to those in other tACS studies – the main risk being that procedures or equipment may fail allowing a metal electrode to come in direct contact with the skin. To ensure that this did not happen all tACS procedures were carried out by a team of two researchers. The first researcher cleaned the scalp, positioned the electrodes and then checked that all cables were correctly connected. The second researchers then performed an independent safety check of all electrodes and equipment. During stimulation one researcher operated the tACS equipment and ran the experiment while the other researcher monitored the electrodes to ensure that everything stayed in place.

#### Tremor measurements

**Figure 1:**
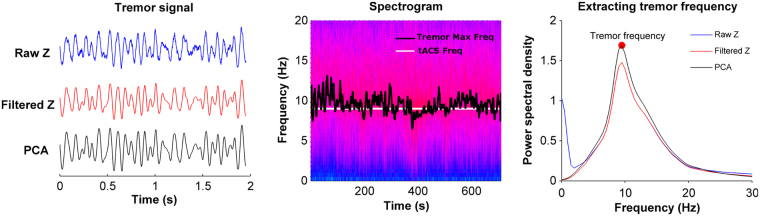
Tremor signal processing and tremor frequency extraction. The left panel shows a 2 second segment of the raw accelerometer data from the z-axis (blue), its corresponding filtered segment (red) and principal component (black). Note the principal component signal combines data from all three accelerometer axis. The middle panel shows the spectrogram of the tremor calculated for during one complete 12 minute session with the black line denoting the instantaneous maximum tremor frequency and the white line showing the tACS frequency. The right panel shows the power spectral density average over the complete 12 minutes for each of the signals shown in the left panel. The PCA signal was used to extract tremor frequency and was also used in all further tremor phase entrainment and amplitude modulation analysis.

Subjects were seated on a chair and instructed to rest the wrist of their dominant hand on a table with fingers extended. A 15 g weight was attached to the middle finger. This induced a measurable postural physiological tremor in all subjects. A triaxial accelerometer (ADXL335, Analogue Devices, Norwood, MA) was attached to the middle finger. Accelerometer data was digitized (1024 Hz) on the aforementioned data acquisition card, and stored for offline analysis. Using custom written MATLAB software, each accelerometer axis was bandpass filtered (3–30 Hz) using a second order Butterworth filter. Principal component analysis was applied, and the first component extracted. An example segment of tremor data is shown in Fig. 1 to illustrate the effect of the various signal processing steps. This was similar to the approach used by Mehta *et al*. to extract tremor data from the accelerometer signal^11^. On average this component explained 88.6 ± 5.7% of the variance (80.0 ± 9.1% in Experiment 2). This waveform was used in all further analyses and is referred to as the tremor signal.

#### Phosphene measurements

To familiarize the subject with phosphenes, tACS amplitude at one site was increased from 0 mA in 0.2 mA steps. Before starting stimulation we described phosphenes as either a flickering light or flickering in their peripheral vision and instructed them to look out for this effect. After each increase, the subject was asked if they perceived phosphenes or noticed any other effect on their vision. This continued until the subject perceived phosphenes or the maximum peak-amplitude of 2 mA (i.e. 4 mA peak-to-peak) was reached. This procedure was repeated for the other site to ensure that the subject had experienced and became familiar with phosphenes at both sites before making any phosphene measurements.

To make a phosphene threshold measurement, amplitude was slowly increased until the subject perceived phosphenes. Once they reported phosphenes, amplitude was slowly decreased until phosphenes disappeared, and then increased again in 0.1 mA steps until phosphenes were just perceivable. This was noted as the phosphene threshold and the procedure repeated for the other site. Site order (MC or PFC first) was counterbalanced across subjects.

To make a phosphene intensity measurement tACS amplitude was then slowly increased in 0.2 mA steps towards a maximum peak-amplitude of 2 mA (i.e. 4 mA peak-to-peak). Subjects were instructed that they would feel the stimulation but it should not be uncomfortable. If tACS was uncomfortable, amplitude was reduced. This was repeated for both sites and the lowest comfortable amplitude (of either stimulation site) was then used on both sites throughout the rest of the experiment. After having experienced stimulation on both sites, the subject was asked to rate phosphenes intensity at the lowest comfortable amplitude (same amplitude on both sites) using a visual analog scale (VAS) from 0 to 10.

#### Experimental protocol

Before stimulation a 5 minute period of tremor was recorded to extract individual tremor frequency by calculating the maximum power spectral density averaged over time^11^ (Fig. 1, right panel). For all subjects, tACS was a sinewave with a frequency equal to this individual tremor frequency. If the average tremor frequency during the experiment differed from the stimulation frequency by more than 2 Hz, the subject was excluded. Electrode impedance was below 5 kΩ. Each subject completed two 12 minute sessions with a 10 minute break. During the session, the subject was instructed to keep their hand in the tremor inducing posture and accelerometer data was continuously recorded. The first session began with 30 s of stimulation OFF (i.e. no stimulation), followed by 1 minute of MC stimulation, then 30 s OFF and then 1 minute of PFC. Four repetitions of this 3 minute sequence comprised one 12 minute session. The second session was the same but started with PFC.

#### Tremor Data Analysis – Phase Entrainment and Amplitude Modulation

**Figure 2:**
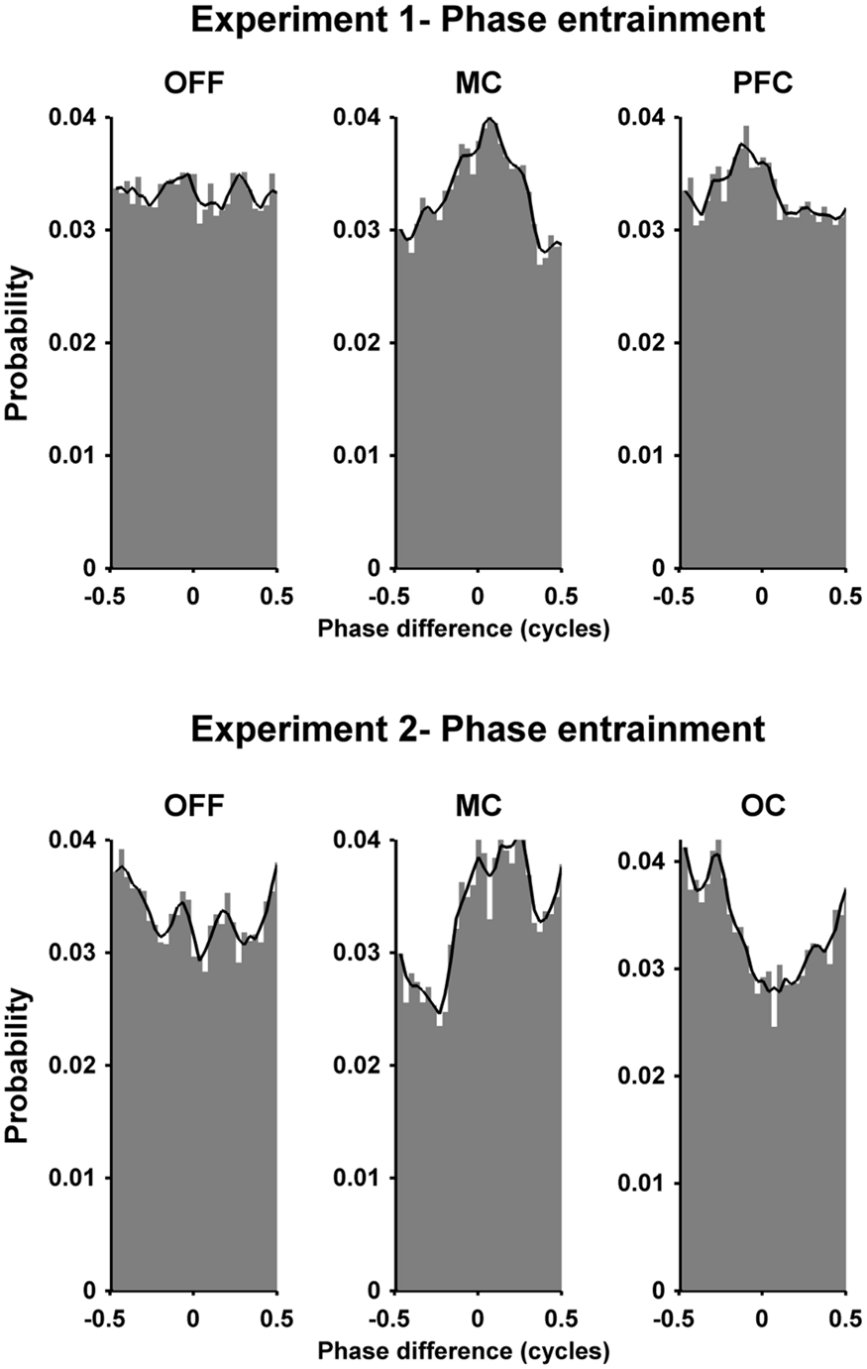
Phase-difference probability histograms illustrating how tACS entrains physiological tremor. The first row shows an example subject from Experiment 1 (subject 1.2). During the OFF condition each phase-difference occurs with approximately equal probability. However, during the motor cortex (MC) and prefrontal cortex (PFC) stimulation the probability of one particular phase-difference occurring is increased, indicating phase entrainment between the tACS and tremor. The black line is smoothed version of the maximum values from each bin and servers only to highlight the histogram contour. Similarly, the second row shows an example subject from Experiment 2 (subject 2.1) for the three conditions OFF, MC and occipital (OC) respectively. Again, during OFF condition the each phase-difference occurs with approximately equal probability. However, during the MC and OC conditions the probability of one particular phase-difference occurring is increased, indicating entrainment between tACS and tremor.

Two oscillators are said to be entrained when their phases are aligned^17^. Entrainment can occur between oscillators that are weakly coupled, allowing one oscillator to impart small amounts of energy to another. Thus, one measure of the effect of tACS (oscillator one) on physiological tremor (oscillator two) is the amount of entrainment. To calculate this, we performed a Hilbert transform on the tACS and tremor waveforms to extract instantaneous phase. The phase signals were subtracted and a 30 bin phase-difference histogram constructed. This was normalized by the total number of samples, yielding a phase-difference probability histogram (Fig. 2). If there is no entrainment, phase-differences are uniformly distributed. If there is perfect entrainment (i.e. constant phase-difference) all the phase-differences appear in one bin. To quantify this we calculated the phase lock value (PLV),

1 $${\rm{PLV}}=|\,\sum _{b}{R}_{b}{e}^{i\theta b}\,|$$

where ${\theta }_{b}$ and ${R}_{b}$ are the center and the amplitude of bin *b*. Thus, PLV = 0 indicates no entrainment (a uniform histogram) and PLV = 1 indicates complete entrainment (all phase-differences in one bin). If tACS entrains tremor, PLV during MC or PFC stimulation will be greater than zero. To determine if this deviation from zero was significant we calculated PLV for stimulation OFF. To do so we constructed a simulated tACS waveform during the OFF periods by copying the tACS waveform from the MC period. We then calculated the same phase-difference histogram between tremor during the OFF periods and the simulated tACS, and the same PLV. This gives a baseline PLV between two uncoupled oscillators (i.e. simulated tACS and tremor during OFF). This allowed us to test for significant differences in the PLV during MC, PFC and OFF.

To assess the effect of tACS on the physiological tremor amplitude a Hilbert transform was performed on the tremor signal to extract instantaneous tremor amplitude (i.e. the tremor amplitude envelope). For each time sample, amplitude was binned according to the corresponding phase-difference as calculated above^11^. For each phase bin the mean tremor amplitude and the 95% confidence interval was calculated and converted to a percentage using the mean across all bins as reference. This yielded tremor amplitude modulation as a function of phase-difference. The function essentially shows the average tremor amplitude that occurred at each phase difference. The 100% line indicates the average tremor amplitude for all phase differences (i.e. average tremor amplitude during that particular tACS condition). A flat function, where all values are close to 100%, means tremor amplitude was unaffected by the phase-difference between tACS and tremor. A deviation away from 100% means that this particular phase-difference was associated with higher or lower tremor amplitude (i.e. an indication that tACS causes tremor amplitude modulation). Similarly to phase entrainment, tremor amplitude modulation was quantified by calculating a PLV (see Eq. 1), except now the each bin value refers to the mean amplitude tremor. The maximum and minimum points on the tremor amplitude modulation function were used to calculate an average change in tremor amplitude.

#### Statistics

Statistics were performed using MATLAB with a significance level α = 0.05. A two-sided Wilcoxon signed-rank test compared scores for phosphene intensity and thresholds between MC and PFC. For phase entrainment and amplitude modulation, the effect of the condition (MC, PFC or OFF) on the PLV was tested with a non-parametric Friedman test. If significant, a post-hoc analysis was conducted with a two-sided Wilcoxon Signed-Rank test between all pairs of the three conditions. p-values were adjusted for multiple comparisons with a Bonferroni correction. To examine the relationship between tremor phase entrainment and amplitude modulation we first made scatter plots of phase entrainment PLV versus amplitude modulation PLV, where each individual session was shown as one data point. We then used a linear mixed model to predict amplitude PLV using phase PLV as a fixed effect and subject as a random effect. This tested if there was a significant relationship between amplitude PLV and phase PLV (MATLAB, Amplitude PLV ~ Phase PLV + (1|Subject)).

### Experiment 2

#### Subjects

Thirteen healthy volunteers participated in Experiment 2 (4 females; age 24 ± 5). All were right-handed except for one (self-reported). Four subjects who completed Experiment 1 also completed Experiment 2. The experiment was approved by the Medical Ethics Committee UZ KU Leuven/Research (S57869) and was performed in accordance with the relevant guidelines and regulations (clinicaltrials.gov registration number NCT03337334, date 08/11/2017). Informed consent to participate in the experiment was obtained from all healthy volunteers.

#### tACS procedure

Experiment 2 compared the effects of focused MC tACS to focused OC tACS. A set of custom made 4 × 1 gel-filled cup-electrodes were used to create focused stimulation. Cup-electrodes had a 5 ml volume and were constructed from 2 cm diameter plastic cylinders mounted in an EEG cap (EASYCAP GmbH, Germany). The cup-electrodes were filled with 5 ml of electrode gel (Signa Gel, Parker Labs, New Jersey) before an EEG AgCl ring electrode (EASYCAP GmbH, Germany) was fastened into the cup. To target MC the stimulating electrode was located at C3 (C4 for left-handed) with the return electrodes at C1, C5, CP3 and FC3 (C2, C6, CP4 and FC4 for left-handed). Modeling studies from Experiment 1 indicated that the maximal electric field on the cortex was approximately 2 cm lateral to the unfocused MC electrode located between C1 and C3 on the scalp. Therefore, a more lateral location was chosen for the MC focused montage, which had a maximal electric field directly under the center electrode. To target OC the stimulating electrode was located at POz with the return electrodes at Oz, Pz, PO4 and PO3. Before placing the electrodes, the scalp regions under the electrodes were anesthetized using EMLA cream (5%, AstraZeneca, Belgium)^18^. This allowed for stimulation at higher amplitudes without causing discomfort.

#### Tremor measurements

Same as in Experiment 1, except for one detail. As described below in Experiment 2, we recorded 6 minutes of OFF, 3 minutes of MC and 3 minutes of OC per 12 minute block. Therefore, to ensure that the same amount of time was used to calculate the PLV for each condition, only the last 30 seconds of each 1 minute block of the OFF condition was used in the analysis.

#### Phosphene measurements

Same as in Experiment 1. However, tACS amplitude was increased to 5 mA.

#### Experimental protocol

The protocol was similar to Experiment 1: Each subject completed three 12 minute sessions with 10 minute breaks. Sessions began with 1 minute of stimulation at MC or OC, followed by 1 minute of stimulation OFF, followed by 1 minute of stimulation at OC or MC, followed by 1 minute of stimulation OFF. Three repetitions of this 4 minute sequence comprised one 12 minute session. The second session followed the same protocol but started with the other site. Session 3 followed the session 1 protocol.

Tremor data analysis and statistics were the same as in Experiment 1.

#### Electro-anatomical Computational Model

A human head computational model was generated using a multiple-step approach based on MRI images from one subject who did not participate in the tACS experiment. The purpose of the model was to make a general quantitative comparison between the electric field distributions in the brain from the focused and unfocused montages. This study was approved by the Medical Ethics Committee UZ KU Leuven/Research and was performed in accordance with the relevant guidelines and regulations. Informed consent to participate in the experiment was obtained from the subject. First, T1- and T2-weighted MRI images were acquired, with voxel sizes (0.98 × 1.2 × 0.98 mm^3^) and (0.98 × 0.98 × 2 mm^3^) respectively. Different tissues were segmented using SimNIBS^19,20^ and corresponding electrical conductivity values (σ) were assigned: skin 0.465 S/m; skull 0.01 S/m; cerebrospinal fluid 1.65 S/m; gray matter 0.27 S/m; and white matter 0.126 S/m^21–25^. Surface meshes of segmented tissues were exported into ScanIP 7 (Simpleware Ltd, Exeter, UK), where the different electrodes were added and tetrahedral meshes generated.

Four different electrode configurations (2 from each Experiment) were simulated. Since the model includes only the head, the extra-cephalic electrode was modeled as a large electrode covering the base of the head. This assumes that currents are equally distributed along the base of the head for these montages. All electrodes were modeled as thin 1 mm layer with 1.4 S/m for sponge electrodes^21^ and 0.3 S/m for gel-filled cup-electrodes^26^. Tetrahedral meshes were imported into COMSOL multiphysics 5 (COMSOL, Inc., Burlington, MA) where electric field (E) and current density (J) were calculated by solving Laplace’s equation,

2 $$\nabla \cdot {\rm{\sigma }}\nabla {\rm{\phi }}=0\,$$

$$E=|\nabla {\rm{\phi }}|$$

$$J={\rm{\sigma }}|{\rm{E}}|$$

with ${\rm{\phi }}$ representing the electrical potential. This assumes a quasi-static approximation of Maxwell’s equations, valid for alternating electric fields in the brain with frequencies <1 MHz^27^. Boundary conditions were set to have a positive current at the anodic electrode and a negative current at the cathodic electrode(s) with peak-amplitude equal to average peak-amplitude in each experiment.

Two metrics were extracted from the simulations to compare electric field magnitude: The first was the maximum electric field magnitude (E_max_). However, E_max_ can be skewed by a few high-magnitude voxels. Therefore, a second metric which takes the average electric field magnitude for the 5% of voxels with the strongest field was calculated (E_max5%_). To quantify field spatial spread (i.e. a measure of focality) we calculated the half-value volume percentage^28^. This metric represents the percentage of brain volume with a magnitude higher than half E_max5%_. To do this we simply find the volume of brain with a magnitude higher than half E_max5%_ and divide this by the total volume of the brain. For a field that is distributed over a larger volume of brain, the half-value volume percentage will be higher than for a field that is distributed over a smaller volume of brain.

To aid comparison of our results with more standard tACS amplitudes and to gain understanding of the effects of focusing on the electric field strength in the cortex, the model was also calculated using 1 mA tACS for both the focused and unfocused montages.

## Results

### tACS procedures

#### Experiment 1

All subjects completed Experiment 1. Average tACS peak-amplitude was 1.925 ± 0.169 mA (equivalent to current density of 0.077 ± 0.007 mA/cm^2^ under the stimulating electrode and 0.019 ± 0.007 mA/cm^2^ under the return electrode) and average tremor frequency was 9.0 ± 0.5 Hz. The mean frequency difference between stimulation and tremor was 0.51 ± 0.42 Hz. No subjects showed a difference >2 Hz. The most common side-effects were phosphenes, metallic taste and a tapping or tingling feeling on the scalp. Inspection of the skin under the electrodes after the experiment showed no signs of irritation in any subjects.

#### Experiment 2

Three subjects were excluded from Experiment 2. One subject reported feeling dizzy after 3 minutes of tACS. After 10 minutes rest the subject felt ok but did not continue. Follow-up the next day revealed no lasting side-effects. Another subject felt no stimulation during the sessions, a post-experiment check showed that gel had leaked from the central cup-electrode causing bridging with a neighboring electrode. This probably caused most current to be shunted by the gel. A third subject could not maintain a constant tremor frequency, meaning that tremor frequency differed by 4 Hz from the stimulation frequency. This subject’s data are shown in the supplementary results together with a brief discussion of the effects of applying tACS at a frequency other than the tremor frequency. Of the remaining 10 subjects, the mean frequency difference between stimulation and tremor was 0.20 ± 0.26 Hz. Average tACS peak-amplitude was 4.45 ± 0.65 mA (equivalent to current density of 1.416 ± 0.2 mA/cm^2^ under the stimulating electrode and 0.354 ± 0.05 mA/cm^2^ under each of the return electrode) and average tremor frequency was 8.9 ± 0.6 Hz. Subjects reported a tapping of tingling feeling on the scalp but no phosphene or taste side-effects. Inspection of the scalp after the experiment showed no signs of stimulation related irritation.

### Tremor measurements

#### Experiment 1

**Figure 3:**
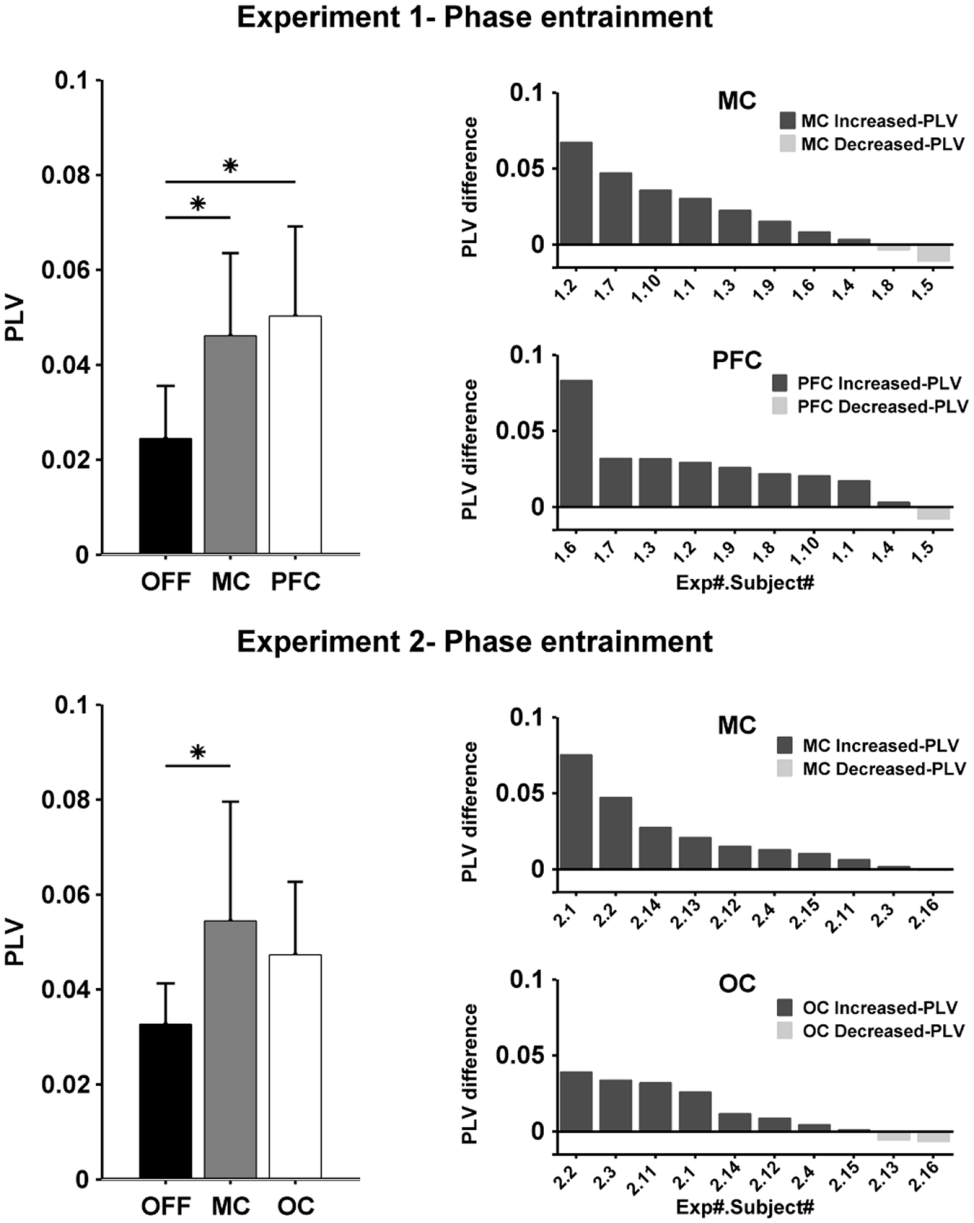
Group data comparing tremor phase entrainment for the different conditions and experiments. The left column shows the mean and the standard deviation of the phase locking value (PLV, calculated from the phase-difference histograms in Fig. 2) at the group level for the different stimulation conditions during Experiment 1 (upper panel) and Experiment 2 (lower panel). The results show a significant increase in the PLV for both stimulation sites in both experiments. The right column shows the difference between the PLV for each stimulation site compared to stimulation OFF for each subject in descending order. Bars with dark color represent subjects that show an increase in PLV compared to OFF, while bars with light color represent subjects that show decrease in the PLV compared to OFF.

Figure 2, top row, shows a phase-difference probability histograms for one subject. The histogram during OFF is relatively flat and close to 0.033 (expected for a uniform probability distribution with 30 bins). As expected, there was no preference for a particular phase-difference since the simulated tACS waveform was not coupled to the tremor. The MC and PFC histograms show an increased probability for one particular phase indicating coupling between tACS and tremor, leading to entrainment. The black lines show the smoothed bin values and serve only to highlight the histogram contour. Figure 3, upper left, shows the PLVs quantifying the uniformity of the histograms for all subjects. Mean PLV was 0.023 during OFF, increasing to 0.044 for MC and 0.048 for PFC. The Friedman test showed a statistically significant effect of condition on PLV (χ^2^(2) = 7.4, p = 0.0247). Post-hoc analysis with Wilcoxon signed-rank test showed a significant difference between MC and OFF (W = 4, p = 0.0137) and a significant difference between PFC and OFF (W = 2, p = 0.005). There was no significant difference between MC and PFC (W = 27, p = 1). Figure 3, upper right panels, shows each subjects increase/decrease in the PLV between MC or PFC and OFF. The number of healthy volunteers showing an increase in phase entrainment compared to OFF was 8 for MC and 9 for PFC. Data for each individual subject and each session are shown in supplementary results.

**Figure 4:**
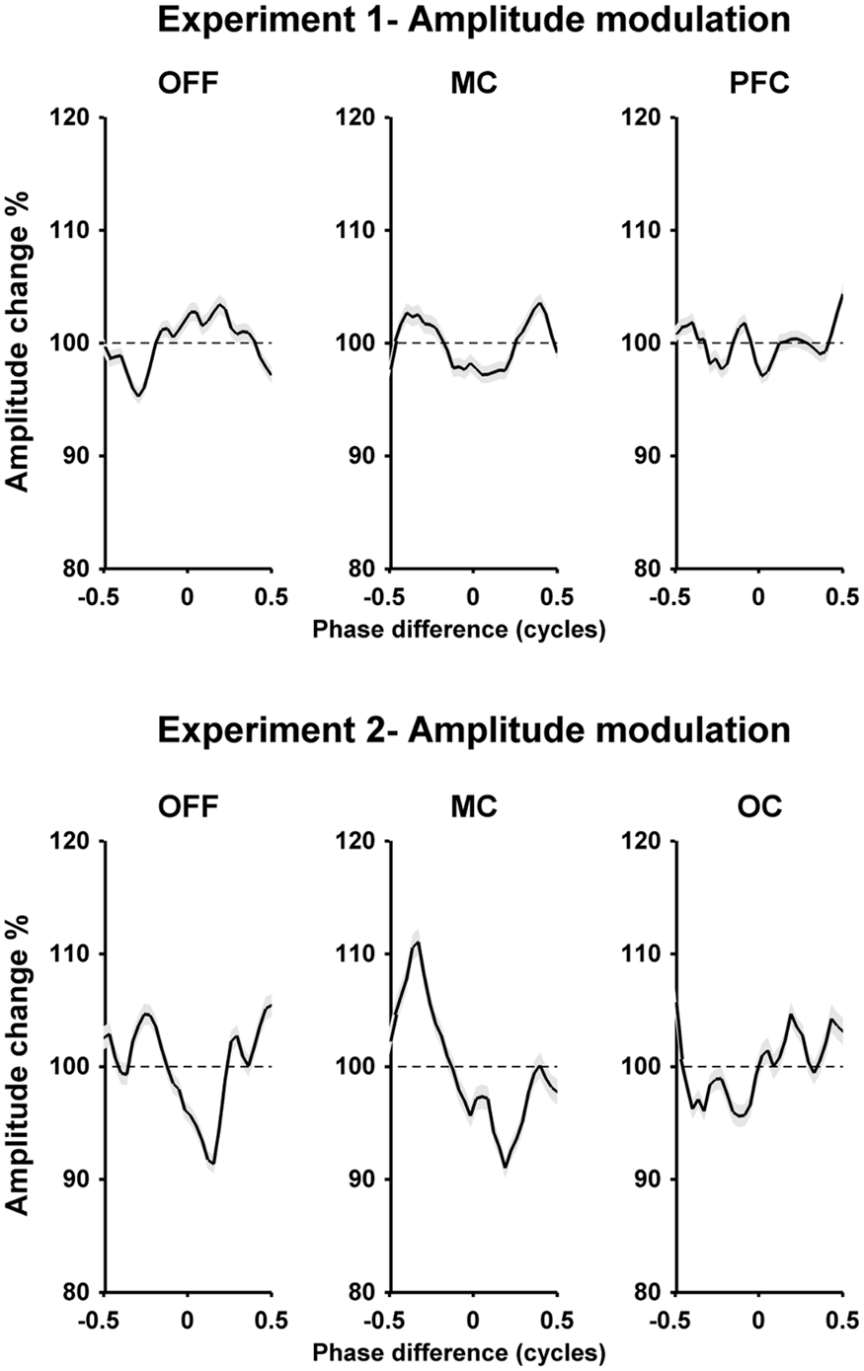
Tremor amplitude modulation functions illustrating the effect (if any) of tACS on physiological tremor amplitude. For each panel, the x-axis shows the phase-difference between tremor and tACS and the y-axis shows the average tremor amplitude that occurred at that phase-difference normalized across all phase-differences for that condition and then expressed as a percentage. The gray band represents the 95% confidence interval. The top row shows an example subject from Experiment 1 (subject 1.4). For each condition - OFF, motor cortex (MC) and prefrontal cortex (PFC) - the amplitude modulation function is relatively flat indicating the lack of a relationship between the occurrence of particular phase and a modulation in tremor amplitude. Similarly, the second row shows an example subject from Experiment 2 (subject 2.4) for the three conditions OFF, MC and occipital (OC) respectively. During OFF condition the amplitude modulation function is relatively flat. However, during the MC and OC condition there appear to be small modulations in tremor amplitude associated with the occurrence of a particular phase-difference.

**Figure 5:**
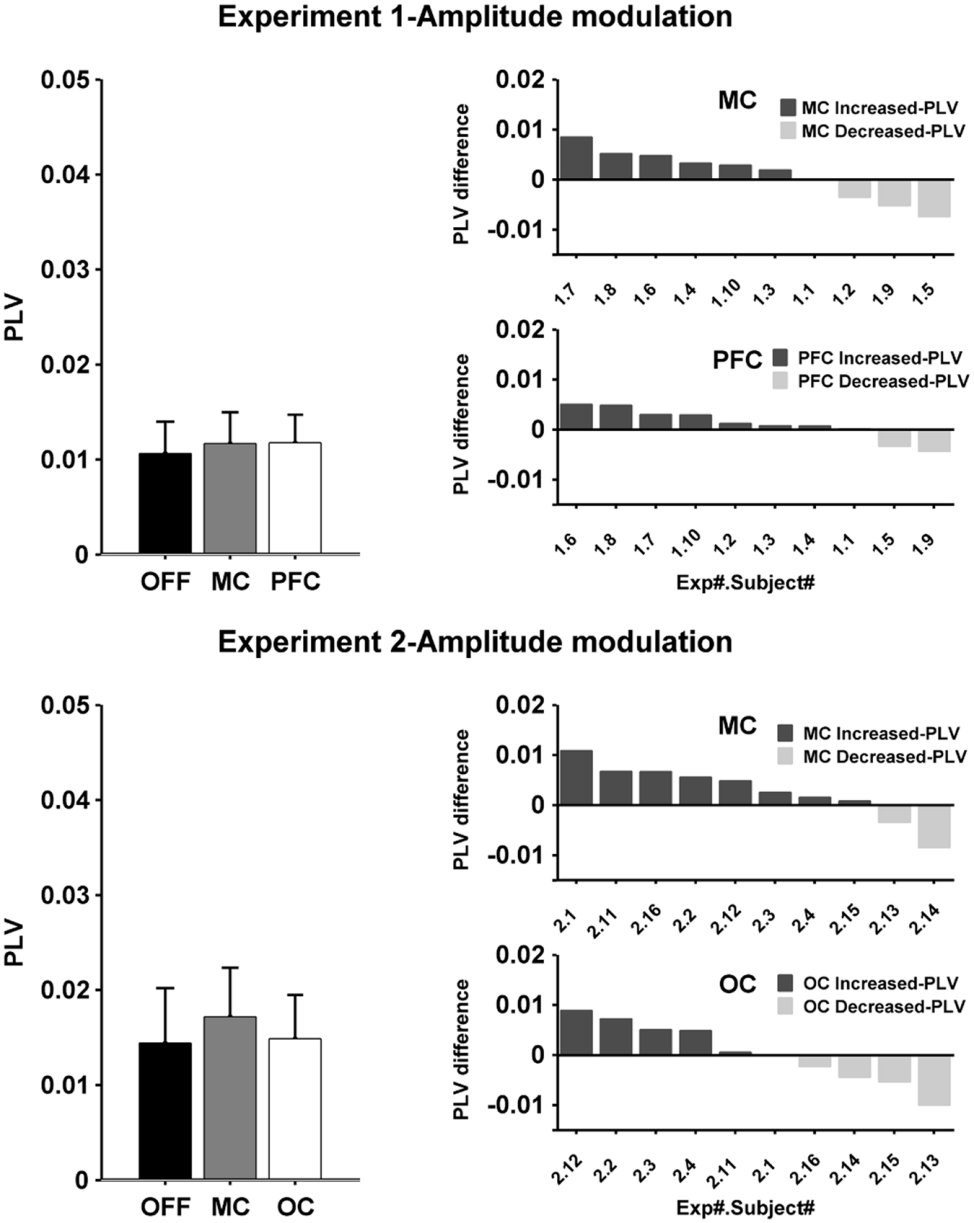
Group data comparing tremor amplitude modulation for the different conditions and experiments. The left column shows the mean and the standard deviation of the phase locking value (PLV, calculated from the amplitude modulation functions in Fig. 4) at the group level for the different stimulation conditions during Experiment 1 (upper panel) and Experiment 2 (lower panel). The results show a no significant change in the PLV, except for a significant difference between the OFF and MC condition in Experiment 2. The right column shows the difference between the PLV for each stimulation site compared to stimulation OFF for each subject in descending order. Bars with dark color represent subjects that show an increase in PLV compared to OFF, while bars with light color represent subjects that show decrease in the PLV compared to OFF.

Figure 4, top row, shows data from one subject where unfocused tACS had little effect on tremor amplitude modulation. Figure 5, upper left, shows the mean tremor amplitude modulation PLV for OFF (0.0122), MC (0.0149) and PFC (0.0137). A Friedman test found no significant effect of condition on amplitude modulation PLV (χ^2^(2) = 5, p = 0.0821). The average change in tremor amplitude were 7.85% for OFF, 7.58% for MC and 7.24% for PFC.

#### Experiment 2

Figure 2, bottom row, shows an example of the phase-difference probability histograms for one subject during OFF, MC and OC. The patterns are similar to Experiment 1: the OFF histogram is flat indicating no phase entrainment while the MC and OC histograms show an increased probability for one particular phase-difference. Figure 3, bottom left, shows the PLV for the three different conditions. Mean PLV was 0.036 during OFF, increasing to 0.054 for MC and 0.047 for OC. The Friedman test showed a statistically significant effect of condition on PLV (χ^2^(2) = 9.8, p = 0.0074). Post-hoc analysis with Wilcoxon signed-rank test showed a significant difference between MC and OFF (W = 1, p = 0.011) and no significant difference between OC and OFF (W = 7, p = 0.111). There was no significant difference between MC and OC (W = 39, p = 0.275). Figure 3, bottom right, shows that the number of healthy volunteers with an increase in phase entrainment compared to OFF was 9 for MC and 8 for OC. Data for each individual subject and each session are shown in supplementary results.

Figure 4, bottom row, shows data from one subject where focused tACS appears to show a small effect on tremor amplitude modulation. Figure 5, bottom left, shows the mean tremor amplitude modulation PLV values for the OFF (0.016), MC (0.0185) and OC (0.0166). While some individual subjects appeared to show small increase in amplitude PLV between the OFF and MC or OC conditions, at the group level a Friedman test found no significant effect of condition on amplitude modulation PLV (χ^2^(2) = 2.4, p = 0.301). The average percentage change in tremor amplitude was 9.71% for OFF, 9.8% for MC and 9.18% for OC.

### Relationship between phase entrainment and amplitude modulation

The previous analysis shows that tACS did not significantly affect tremor amplitude in any condition at the group level. However, as is evident from the individual data (see right panels in Figs 3 and 5), there is a large variation in the effect size between subjects. Therefore, we wanted to know if subjects who showed a larger effect on tremor phase entrainment also showed a larger effect on tremor amplitude modulation.

**Figure 6:**
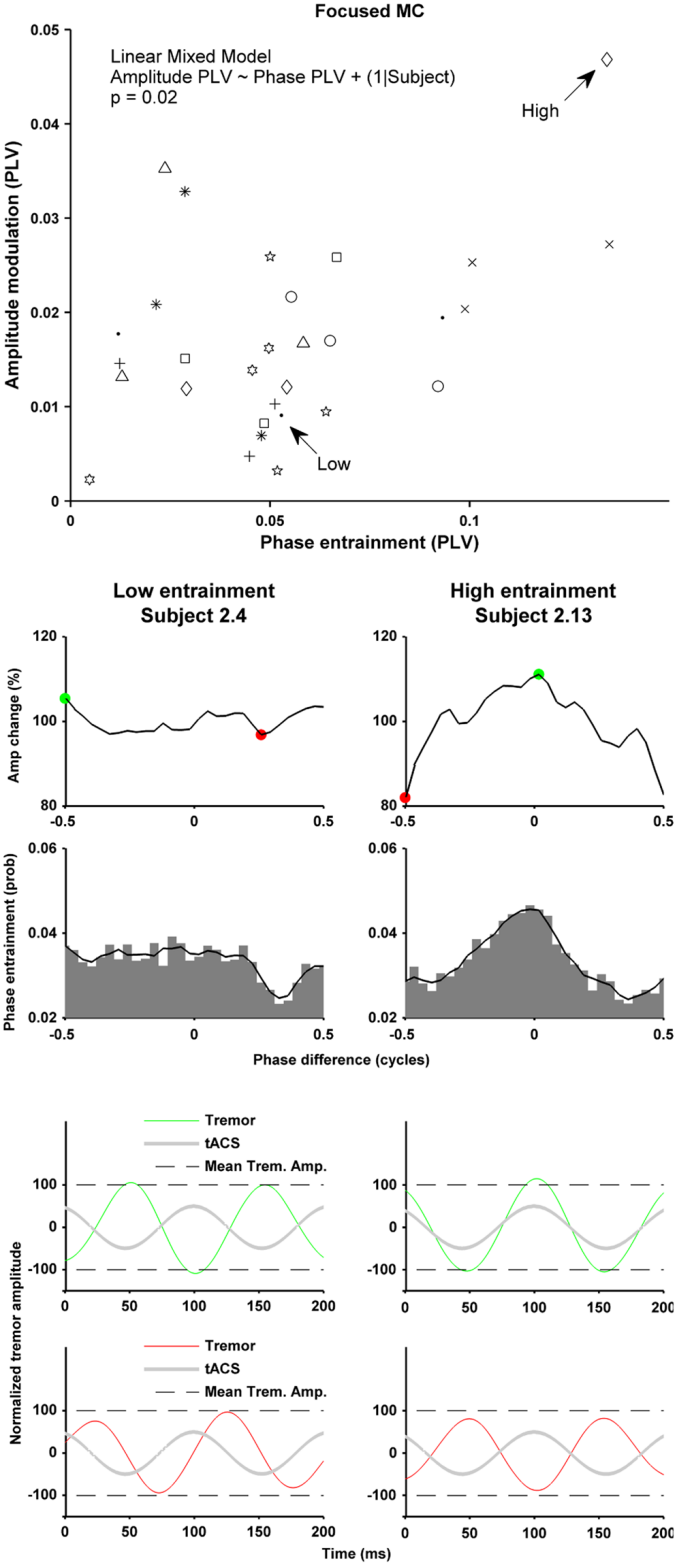
Relationship between tremor phase entrainment and tremor amplitude modulation for the focused MC condition. The scatter plot in the top panel shows the phase entrainment PLV and amplitude modulation PLV for each of the three sessions for each subject (same symbols) during the MC condition in Experiment 2. Linear mixed model analysis showed a significant relationship (significant effect of the fixed effect, phase PLV, on the amplitude PLV, p = 0.024), indicating that subjects with higher phase entrainment also had higher levels of tremor amplitude modulation. The right column shows data from one subject with high levels of entrainment and the left shows a subject with lower levels of entrainment. The first row shows the amplitude modulation histogram. The second row shows the phase entrainment probability histogram. The last two rows illustrate the same findings using averaged time series data: Green/red lines show the average tremor signal for all occurrences of the phase difference indicated by the green/red dot. Grey line shows the associated tACS signal. Dashed lines indicated the tremor amplitude averaged across the complete condition.

We used linear mixed model analysis to quantify the relationship between phase entrainment PLV and amplitude modulation PLV for each experiment and condition. In summary, we found a significant linear relationship (p < 0.05) between phase entrainment PLV and amplitude modulation PLV for both the focused MC (Estimate = 0.22, standard error = 0.05 and p = 0.024) and OC (Estimate = 0.12, standard error = 0.06 and p = 0.036) conditions. The unfocused MC and PCF conditions did not show a significant linear relationship. Nor did the OFF conditions from both experiments. The most significant linear relationship was found during the focused MC condition in Experiment 2 and is shown in Fig. 6. The complete linear mixed model analysis for all conditions are shown in the Supplementary Results. Each data point on Fig. 6 represents a phase and amplitude PLV value for one of the three sessions collected in the focused MC condition, with the same symbol used for data from the same subject. In general, subjects who showed high levels of phase entrainment also show higher levels of tremor amplitude modulation, and visa-versa.

The right column in Fig. 6 shows an example of a subject who exhibits relatively high levels of both tremor phase entrainment and tremor amplitude modulation. In this subject the most commonly occurring phase difference between tACS and tremor was around 0 cycles (i.e. the peak in the phase entrainment probability histogram). When this phase difference was present tremor amplitude tended to increase. However, when a phase difference of around −0.5 cycles occurred tremor amplitude tended to decrease. It should be noted that even in this subject, the effects on tremor amplitude (and phase entrainment) are small. The two panels at the bottom of the column use averaged time series data to show the same effects: the green line shows the averaged tremor signal from all the occurrences when the phase difference with tACS (grey line) was 0 cycles, and the red line shows the same but for all the occurrences when the phase difference was −0.5 cycles. The small differences in tremor amplitude for these two phase differences are just noticeable – the green tremor signal peaks at 110% (just above the dashed line representing average tremor amplitude during the entire condition), while the red tremor signal peaks at 80% (just below the dashed line)

The left column shows an example of a subject with low levels of phase entrainment and amplitude modulation. Any effect of tACS on tremor phase entrainment and amplitude modulation appear negligible in this subject.

### Phosphene measurements

**Figure 7:**
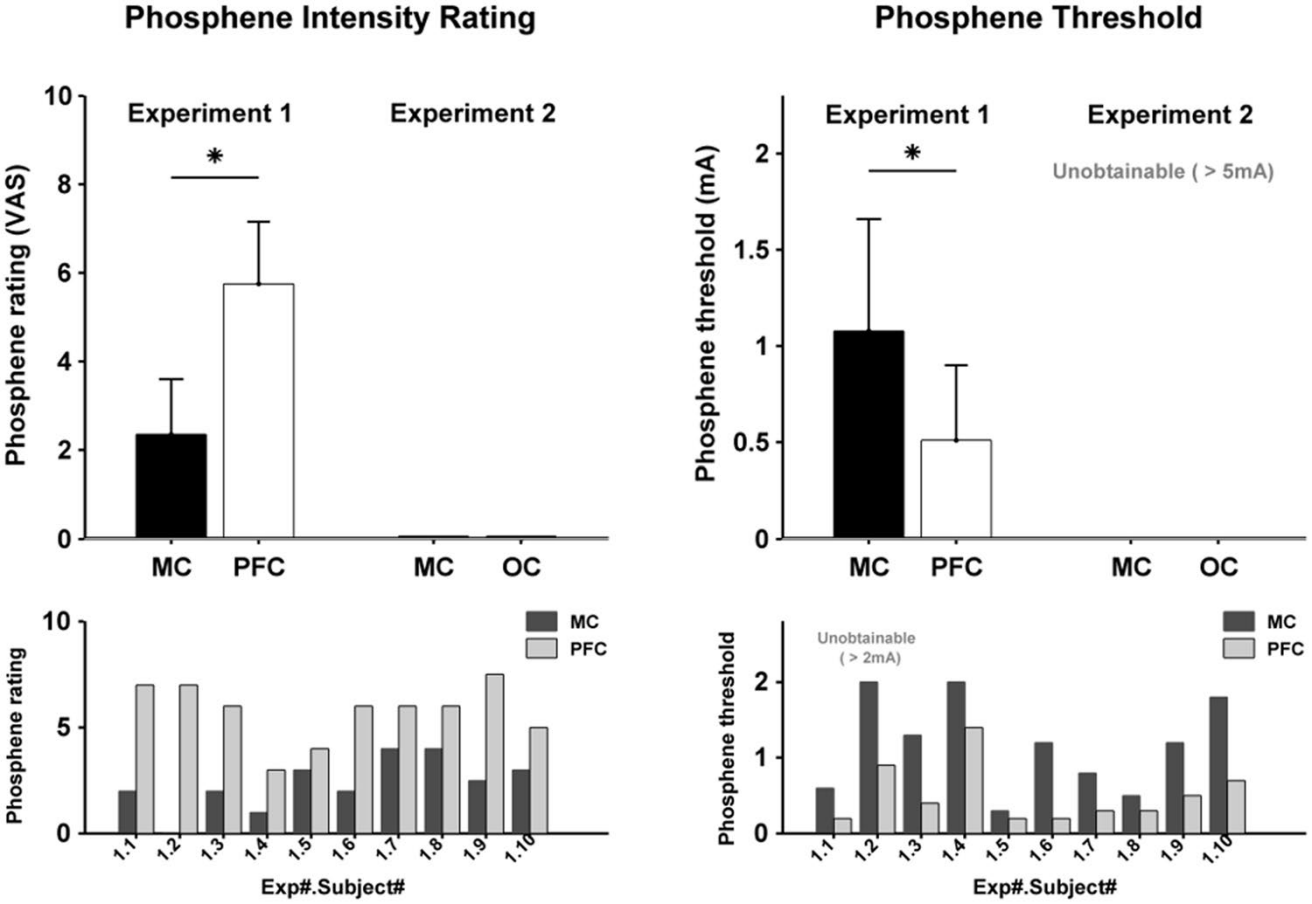
The first row shows phosphene intensity rating and threshold at group level during the different electrodes montages and experiments. The left upper panel shows the phosphene intensity rating based on the visual analog scale (VAS) when tACS was delivered at the maximum amplitude. In Experiment 1 phosphene intensity was rated higher during PFC stimulation than during MC. Using focused tACS in Experiment 2, no phosphenes were perceived at the maximum amplitude. The right upper panel shows the phosphene threshold In Experiment 1. More current was needed to perceive phosphenes for MC than for PFC stimulation. With focused stimulation in Experiment 2 it was not possible to obtain the threshold for phosphene perception. The lower row illustrates the phosphene rating (left lower panel) and phosphene threshold (lower right panel) at individual level during Exp 1.

Figure 7 shows the phosphene measurements. The unfocused montage caused phosphenes in all subjects at both sites (MC and PFC) with the exception of one subject (Subject Number 1.2) who only experienced phosphenes with PFC but not with MC at a maximum of 2 mA (this subject was excluded from the calculation of the average phosphene threshold). In contrast to this, the focused montage caused no phosphenes in any subject at either site (MC and OC). Experiment 1 PFC showed a significantly higher phosphene intensity rating than MC (p = 0.002) with an average VAS of 5.75 for PFC and 2.35 for MC. Phosphene threshold for MC in Experiment 1 was also significantly higher than for PFC (p = 0.0039) with an average peak-amplitude threshold of 1.08 ± 0.58 mA for MC and 0.51 ± 0.39 mA for PFC. Experiment 2 phosphene thresholds were not obtainable at the maximum peak-amplitude.

### Qualitative comparison between focused and unfocused montages

Four healthy volunteers completed both Experiment 1 (subject numbers 1.1, 1.2, 1.3 & 1.4) and Experiment 2 (subject numbers 2.1, 2.2, 2.3 & 2.4) allowing us to make a direct qualitative comparison between the unfocused and focused tACS montages. In Experiment 1 subjects 1.1 and 1.2 showed a relatively large increase in phase entrainment between the OFF and MC conditions, while subjects 1.3 and 1.4 showed a much smaller increase between the same conditions (see Supplementary Results, Individual Phase Entrainment Exp 1: Averaged Data, first 4 plots). Interestingly, a similar pattern was observed with the focused tACS in Experiment 2 with subjects 2.1 and 2.2 showing a larger increase in PLV between the OFF and MC conditions than subjects 2.3 and 2.4 (see Supplementary Results, Individual Phase Entrainment Exp 21: Averaged Data, first 4 plots). Although the total number of subjects is small, these results indicate that the effects of tACS may be subject dependent and that this effect holds across different montages. This is also supported by the correlation analysis (see Fig. 6 and Supplementary Results) where we see that some subjects exhibit high level of tremor entrainment while other exhibit lower levels.

### Electro-anatomical computational model

**Figure 8:**
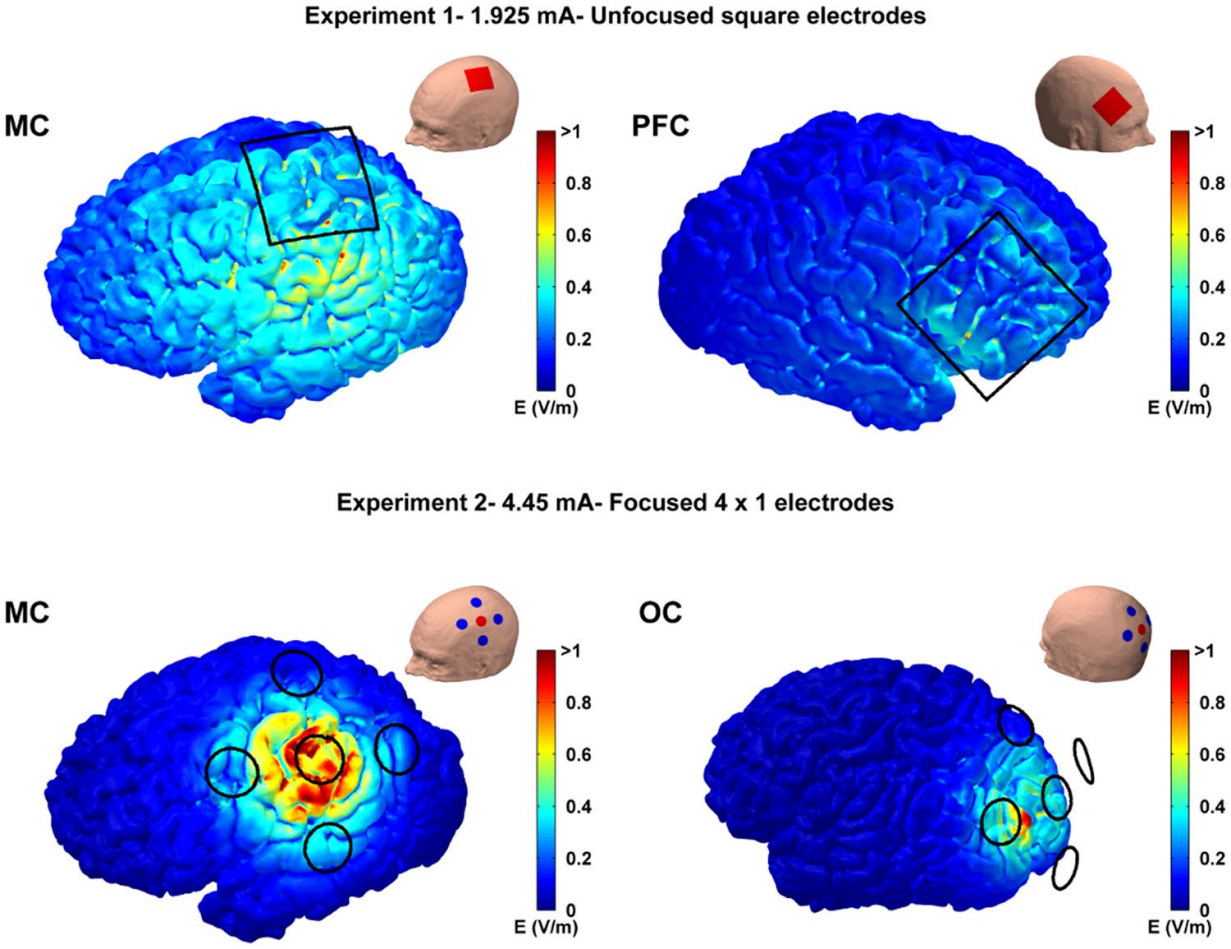
MRI-based electro-anatomical computational model showing the electric field magnitude distribution on the cortical surface for the different stimulation sites and experiments. Black lines represents electrode edges on the scalp. The insets show the orientation of the head and electrode locations for each panel. Anode electrodes are shown in red while cathodes are shown in blue. Top row shows the results for motor cortex (MC) and prefrontal cortex (PFC) tACS in Experiment 1 when the average peak-amplitude was 1.925 mA. The bottom shows the results for motor cortex (MC) and occipital (OC) tACS in Experiment 2 when the average peak-amplitude was 4.450 mA.

Figure 8, top row, shows the cortical electric field for Experiment 1 when 1.925 mA tACS was applied to the MC or PFC. Figure 8, bottom row, shows the same for Experiment 2 when 4.45 mA tACS was applied to the MC or OC. The results show broad electric fields for Experiment 1 and highly focused fields for Experiment 2. In Experiment 1 E_max_ was 1.20 V/m for MC and 0.77 V/m for PFC; while E_max5%_ was 0.45 V/m for MC and 0.29 V/m for PFC. In Experiment 2 E_max_ was 1.48 V/m for MC and 0.92 V/m for OC; while E_max5%_ was 0.41 V/m for MC and 0.28 V/m for OC. The fact that a much higher peak-amplitude is needed with the focused montage to achieve an electric field strength roughly equivalent to the unfocused montages is an expected result as it is already well established, particularly in cochlear implants^29,30^, that near-field focusing reduces electric field strength. The half-value volume percentage (a measure of electric field focality) in Experiment 1 was 46% for MC and 36% for PFC, while in Experiment 2 it dramatically decreased to 6% for both MC and OC.

**Figure 9:**
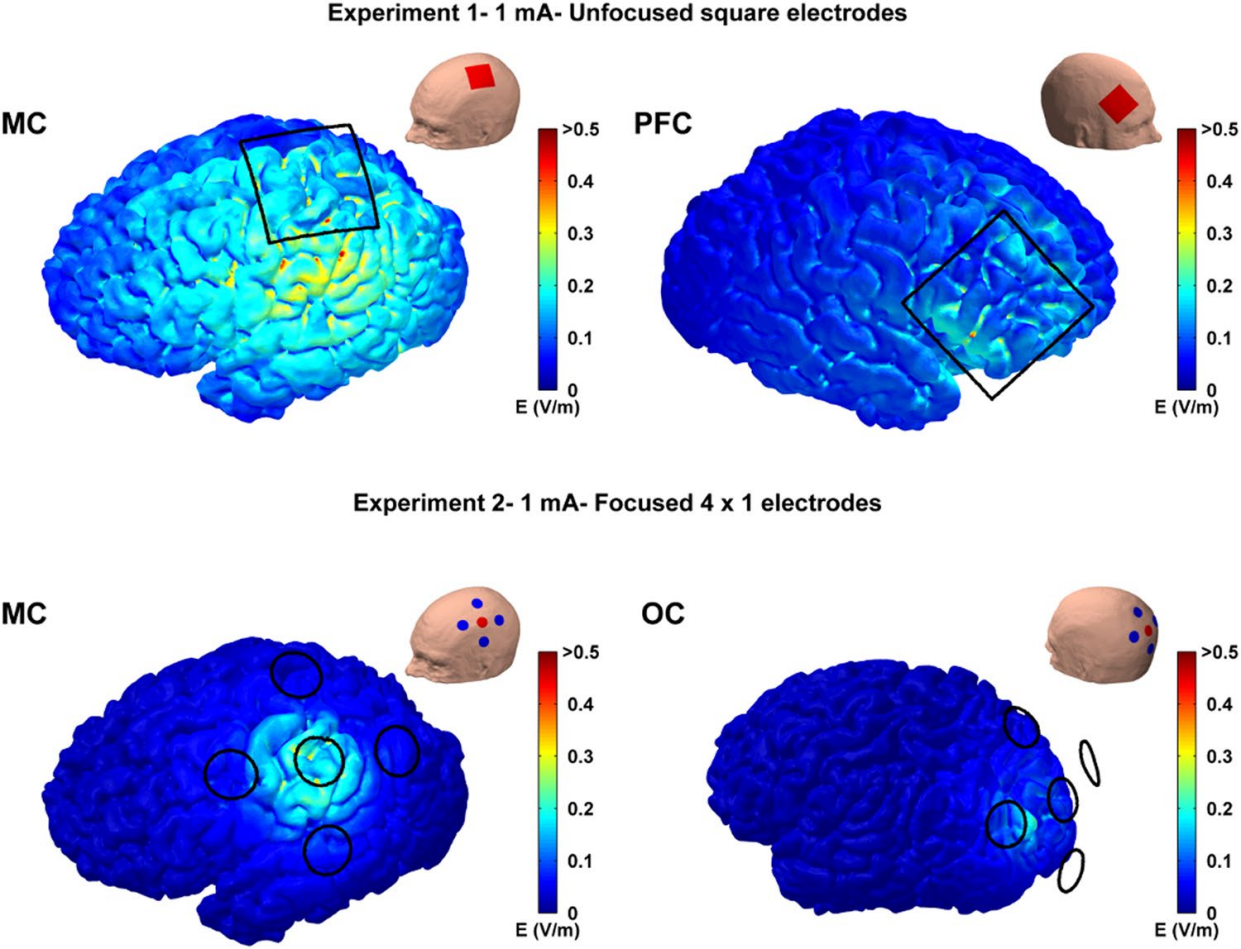
Computational modelling results illustrating the electric field strength reduction during focused stimulation. The electric field distribution was calculated for both experiment montages when the same peak-amplitude current (1 mA) was used. The results show a reduction in the cortical field values with the focused montage compared to unfocused montage. This decrease can be recovered by increasing tACS amplitude (see Fig. 8).

To better understand the effects of focusing on the electric field distribution in the cortex we linearly rescaled the electric field values for each montage to show the field distribution for standard, matched, amplitudes of 1 mA for both the unfocused and focused montages. The results are shown in Fig. 9. Table 1 shows 1 mA unfocused and focused E_max_ values and contrasts these with the E_max_ values achieved in Experiment 1 and 2 using higher amplitude tACS (i.e. 1.925 mA and 4.45 mA respectively). It is clear that focusing the electric field causes a reduction in electric field strength, but that this can be recovered by increasing tACS amplitude for the focused montage.

**Table 1:** Comparison of the maximum electric field strength (E_max_) using focused and unfocused montages with either matched tACS amplitudes at 1 mA or with the tACS amplitudes used in Experiment 1 (1.925 mA) and Experiment 2 (4.450 mA). Focusing the electric field causes a reduction in the electric field strength which can be recovered by increasing tACS amplitude.

	Emax using Unfocusedsquare electrodes	Emax using focused 4 × 1 electrodes
Comparison: 1 mA over MC	0.62 V/m	0.33 V/m
Exp1: 1.925 mA over MC	1.20 V/m	—
Exp2: 4.450 mA over MC	—	1.48 V/m

## Discussion

Our results indicate that it is feasible to use high-amplitude tACS in healthy volunteers. We observed no instances of stimulus related skin irritation. However, we caution that more evaluation is needed before this approach can be widely adopted. We found that high-amplitude tACS could entrain physiological tremor: when averaged across all four tACS conditions (unfocused MC, unfocused PFC, focused MC, focused OC), 85% of volunteers showed an increase in phase entrainment compared to the OFF condition, while only 12.5% showed a decrease in phase entrainment. In line with our hypothesis we found that unfocused PFC tACS causes significantly stronger phosphenes than unfocused MC tACS and that both focused montages cause no phosphenes. We found that both unfocused montages (MC and PFC) caused statistically significant tremor phase entrainment at the group level. However, for the focused montages we found that only MC tACS caused statistically significant phase entrainment at the group level, while OC tACS did not. These findings support our hypothesis that focused MC tACS would cause tremor entrainment while OC tACS would not, and lend support to the idea that focused montages can improve the ability of tACS to target a specific brain region. On the other hand, it is important to highlight the non-significant trend towards increased phase entrainment with the focused OC montage. This appears to suggest that, at least in some subjects, it may not always be necessary to target tACS to a specific brain location to observe an effect.

At the group level we did not observe any statistically significant effects of tACS on tremor amplitude modulation. However, linear mixed model analysis did reveal a statistically significant relationship between phase entrainment PLV and amplitude modulation PLV in the focused MC and OC conditions. This relationship was strongest in the focused MC condition. No significant relationship was found with the unfocused MC and PFC conditions, nor with both OFF conditions. With high amplitude focused tACS we found that subjects who exhibited stronger phase entrainment also showed higher levels of amplitude modulation. Examination of individual phase and amplitude modulation histograms showed that one particular phase difference was associated with a small increase in tremor amplitude, while another phase difference was associated with a small decrease in tremor amplitude. The linear relationship between phase entrainment and amplitude modulation indicates that if we could increase the effectiveness of tACS in some subjects (either by individualized electrode placement or increase tACS amplitudes) we would see a significant effect on tremor amplitude.

In this study electrodes were placed based on 10–20 EEG positions. We did not use individualized anatomy (obtained either from MRI scans or TMS hotspot measurements) to position electrodes. An electrode placement based on individualized anatomy may have reduced variability and increased our effect size. However, we are not aware of any tACS studies to date that specifically show a benefit of using individualized electrode placement.

Our findings are in general agreement with the work by Mehta *et al*.^11^ using 1 mA peak-amplitude tACS. They tested the effect of four unfocused tACS montages and flashing light stimulation on physiological tremor entrainment and phosphene intensity. Each montage had one electrode over left M1 while the return electrode had either a fronto-orbital, contralateral M1, left shoulder or right shoulder location. The flashing light caused significant tremor entrainment but only the tACS montage with the right shoulder return caused significant entrainment. Phosphenes were reported for all montages except for a contralateral M1 return electrode, with a fronto-orbital return receiving the strongest, significant, phosphene rating. However, Mehta *et al*.^11^ found that none of the montages had any effect on tremor amplitude and they did not report any relationship between increased phase entrainment and amplitude modulation.

In Experiment 1 all subjects perceived phosphenes for both the unfocused MC and PFC, except for one subject who did not perceive phosphenes for the MC condition. From these results, it is not possible to say if phase entrainment was caused directly by the electric field modulating neurons in the motor cortex or indirectly through the tACS generated phosphenes. However, in Experiment 2 no subjects reported any phosphenes for either focused MC or OC tACS. Thus, we can conclude that high-amplitude tACS can entrain physiological tremor through a mechanism other than phosphene generation. We observed a statistically significant increase in tremor phase entrainment using the focused MC montage but not with the focused OC montage. Based on these results we can conclude that the oscillating electric field in the brain created by the MC montage most likely contributes to the entrainment of physiological tremor. However, there is a non-significant trend in the focused OC data towards an increase in physiological tremor entrainment. This suggests that, at least in some subjects, it may not always be necessary to specifically target tACS to particular brain region. It may be that inducing a strong enough oscillation anywhere in the nervous system can entrain tremor. Indeed, we already know that flashing a light at tremor frequency (i.e. creating an oscillation in the optic nerve) causes tremor entrainment^11^. Thus, creating an oscillation in a cranial or scalp nerve may also cause tremor entrainment. Alternatively, creating a strong enough oscillation in the visual cortex (i.e. with focused OC tACS) may lead to tremor phase entrainment. These may be one of the mechanisms driving the effects observed with focused OC tACS.

An interesting and important finding from the electro-anatomical computational model was that, when matched for tACS amplitude, a focused montage creates a weaker electric field on the cortex than an unfocused montage (Fig. 9 and Table 1). The effect of focused montages reducing the electric field strength in the brain is in line with previous modeling studies by Datta *et al*.^21,31^. We know from *in-vitro* slice work that weaker electric fields cause less membrane polarization^3^. In line with this we recently demonstrated that the effect of tACS on the rat motor excitability shows an approximately linear dependence on the electric field strength – with stronger electric fields having a stronger effect^32^. Thus, the implication is that focused montages create a weaker electric field in the cortex, leading to less membrane polarization and a weaker tACS effect. Importantly, our computational model results indicate that the electric field strength for the focused montages can be increased by simply increasing the tACS amplitude (Fig. 8 and Table 1). It should be noted that the reduction in electric field strength due to this type of nearfield focusing is an expected result and one that has been demonstrated in a number of different fields^29,30,33^. The results from our two experiments, combined with the safety study shown in supplementary methods, indicate that delivering tACS at these higher amplitudes may be safe. However, further studies involving larger population are needed to confirm this.

We used exactly the same equations and approach to model the tACS electric field distribution as is used to model the electric field for transcranial direct current stimulation (tDCS). We do this because the quasi-static approximation of Maxwell’s equations are valid for alternating electric fields in the brain with frequencies < 1 MHz^27^. This means that our finding that focused tACS montages reduce the electric field strength on the cortex will also apply to focused tDCS montages.

Finally, our results give insight into the mechanism through which tACS generates phosphenes. Some studies suggest phosphenes are caused by direct visual cortex modulation, while others suggest a retinal origin. Experiment 2 shows that high-amplitude, focused, OC tACS did not generate phosphenes in any subjects (although we should point out that we did not use electrode placements based on individual anatomy). While Experiment 1 shows that PFC tACS generated significantly stronger phosphenes than MC tACS. Thus, our results point toward a retinal, and not a visual cortex, origin for tACS phosphenes.

## Electronic supplementary material

Safety report and supplementary results
