# Publisher Correction: Using high-amplitude and focused transcranial alternating current stimulation to entrain physiological tremor

**DOI:** 10.1038/s41598-018-26013-3

**Published:** 2018-05-23

**Authors:** Ahmad Khatoun, Jolien Breukers, Sara Op de Beeck, Ioana Gabriela Nica, Jean-Marie Aerts, Laura Seynaeve, Tom Haeck, Boateng Asamoah, Myles Mc Laughlin

**Affiliations:** 1 ExpORL, Department of Neurosciences, KU Leuven, B-3000 Leuven, Belgium; 2 Division Animal and Human Health Engineering, Department of Biosystems, Faculty of Bioscience Engineering, KU Leuven, B-3000 Leuven, Belgium; 3 Department of Neurology, Laboratory for Epilepsy Research, University Hospitals & KU Leuven, B-3000 Leuven, Belgium; 4 Medical Imaging Research Center (MIRC), KU Leuven, B-3000 Leuven, Belgium; 5 Center for Processing Speech and Images (PSI), Department of Electrical Engineering (ESAT), KU Leuven, B-3000 Leuven, Belgium

Correction to: *Scientific Reports* 10.1038/s41598-018-23290-w, published online 21 March 2018

In the original version of this Article, the author Sara Op de Beeck was incorrectly indexed. This error has now been corrected.

