# Supplementary material for: Using high-amplitude and focused transcranial alternating current stimulation to entrain physiological tremor
Source: Sci Rep. 2018 Mar 21;8:4927. doi: 10.1038/s41598-018-23290-w (PMC5862845; doi:10.1038/s41598-018-23290-w)

Using high-amplitude and focused transcranial alternating current stimulation to entrain physiological tremor

Authors

Ahmad Khatoun, Jolien Breukers, Sara Op de Beeck, Ioana Gabriela Nica, Jean-Marie Aerts, Laura Seynaeve, Tom Haeck, Boateng Asamoah, Myles Mc Laughlin

Supplementary Information

A stand-alone report on the safety and risk assessment on using 10 mA focused tACS

SAFETY REPORT: Risk Assessment of 10 mA Focused tACS

Introduction

Transcranial alternating current stimulation (tACS) is a noninvasive neuromodulation method in which electrodes are attached to the scalp of the patient or healthy volunteer and alternating current (usually a sine wave) is then passed through these electrodes. Most of the current passes via the skin to the return electrode, but a portion passes through the skin, skull and cerebrospinal fluid to enter the cortex and cause neuromodulation. The theoretical risks which arise when using any transcranial brain stimulation method are listed below in order of severity:

- Damage to neural tissue
- Seizure
- Skin burn
- Skin irritation
- Pain (caused by stimulation of nerves in the scalp)
- Tissue heating

As is widely reported in the literature (reviewed in [1]), when tACS is applied at amplitudes of 2 mA for a duration of 20 minutes through large saline soaked sponge electrodes ($5cm^2$ surface area) in an unfocused configuration it is safe, i.e. none of these theoretical risks have been reported to occur (Fig. 1A & B). However, it should be noted that at 2 mA the subject does feel a tingling sensation under the electrode probably caused by stimulation of nerves in the skin. Two main issues with standard tACS are: 1) It produces a broad electric field causing side effects such as visual phosphenes. 2) Because the electric field on the cortex is weak (around 1V/m, compare this with deep brain stimulation where fields of 100 V/m are used [2]), the therapeutic effect is weak and has poor reproducibility between and within subjects. To improve tACS method and overcome these limitation we propose to use stronger (10 mA), focused, tACS.

The aim of this report is to assess the risk of applying focused tACS at 10 mA using small cup gel-filled electrodes ($2.6cm^2$ surface area). This approach has already been used with transcranial direct current stimulation at amplitudes of 2 mA (see http://www.soterixmedical.com/hd-tdcs for commercially available FDA and CE approved devices). In focused tACS the return electrodes are place closer to the stimulating electrode to limit current spread to just the brain area under investigation (Fig. 1E &F). To assess the risks associated with 10 mA focused tACS we conducted a review of all transcranial brain stimulation methods and calculated a number of different stimulation strength metrics. Below we first explain each of stimulation strength metrics and then introduce each of the different transcranial brain

stimulation methods. We then compare stimulation strength between the different brain stimulation methods. Our review found that 10 mA focused tACS would not pose a significant risk to the subject.

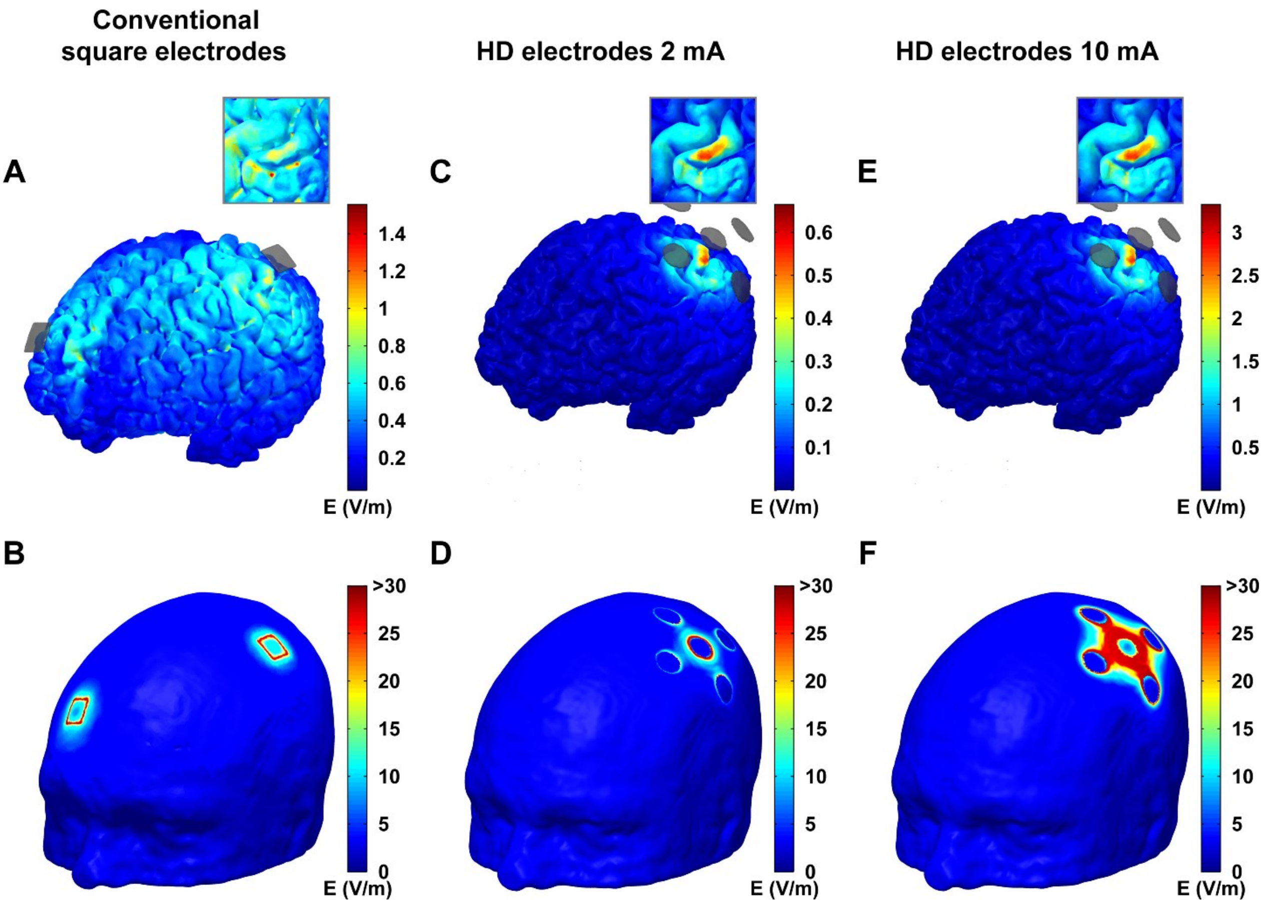

Figure 1. Electro-anatomical model based on segmented MRI data and finite element method. We used this model to estimate the electric field strength on the cortex from different tACS configurations and amplitudes. A & B show the electric field strength on the cortex and skin for conventional 2 mA tACS. C & D show that a configuration of smaller (HD) electrodes can be used to focus the electric field but at the cost of reduced electric field strength. E & F show that increasing tACS amplitude to 10 mA with the focused configuration can produce a stronger, spatially limited, electric field on the cortex. When compared with other transcranial brain stimulation methods, 10 mA focused tACS is still well within the safety limits. Note that because of the large difference in electric field strength, the different panels use different color bar scales

Stimulation Strength Metrics

A wide range of metrics exist to calculate and quantify neuromodulation strength. All these metric depend on the amplitude and duration of the applied current and the electrode surface area. Here we organize the most important metrics based on their point within the stimulation chain (see Fig. 2):

1) At the level of neuromodulation device:

- Current amplitude (mA)
- Phase duration (ms) *(i.e. if pulse train stimulation is used this is the duration of one phase in a biphasic pulse, if sine wave stimulation is used this is the duration of one phase of the sine wave*),
- Charge per phase (mC)
- Dose duration (s) *(i.e. the total stimulus duration)*
- Charge per dose (mC)

Current amplitude, phase and dose duration are know exactly from the device settings. Charge per phase is calculated for a pulse stimulus as phase duration times current amplitude, or for a sine wave stimulus by taking the integral of one phase. Charge per dose is calculated by counting the total number of phases presented to the subject (both negative and positive) and then multiplying by the charge per phase.

2) At the level of the electrode-tissue interface:

- Current density (mA/cm^2)
- Charge density per phase (mC/cm^2)
- Charge density per dose (mC/cm^2)

These metrics depend on the size of the electrode surface area. Current density is calculated by dividing the current amplitude by the electrode surface area. Charge density per phase/dose is calculated by dividing charge per phase/dose by the electrode surface area.

3) At the level of the brain tissue:

- Current density (A/m^2)
- Electric field (V/m)

These metrics define stimulation strength within the brain and are difficult to measure directly. However, they can be estimated using electro-anatomical computational models. Fig. 1 shows data from a finite element model that we implemented based on MRI data which is segmented into skin, skull, cerebrospinal fluid, grey matter and white matter. We used this model to calculate electric field strength and current density of different tACS electrode configurations and strength.

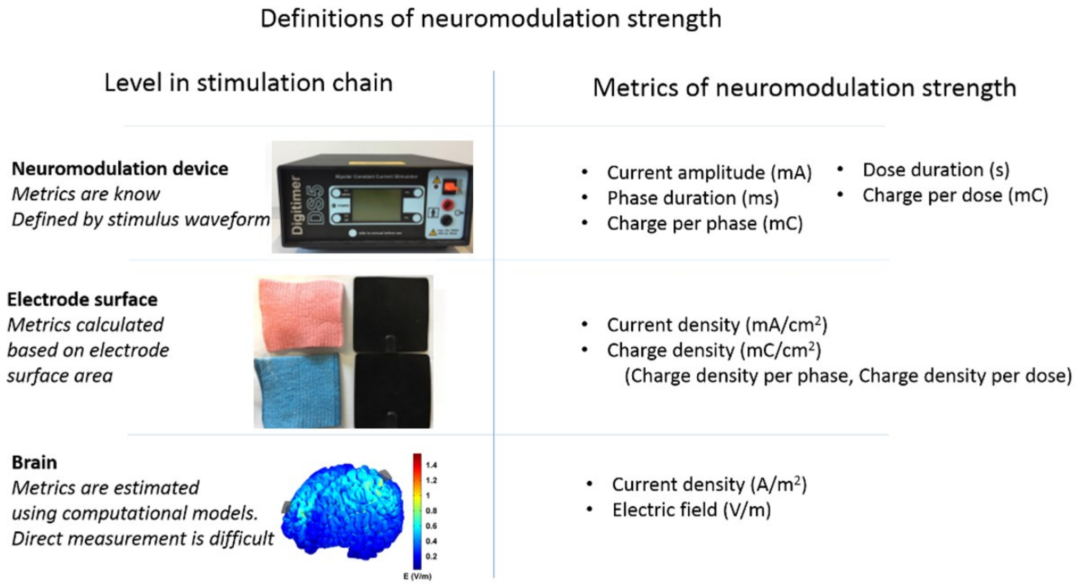

Figure 2 Overview of stimulation strength metric which can be calculated at 3 points in the stimulation chain.

Transcranial Brain Stimulation Methods

Here we list the five most commonly used transcranial brain stimulation methods and briefly describe their application and mechanism of action.

1. Electroconvulsive therapy (ECT)

Application - Treatment of psychiatric disorders
Patient state - General anesthesia
Mechanism - The goal of ECT is to cause a seizure. Stimulation is applied at 2 to 6 times seizure threshold

2. Motor evoked potentials (MEP)

Application - Monitor integrity of motor system during neurosurgery
Patient state - General anesthesia
Mechanism - Suprathreshold stimulation initiates action potentials in motor cortex causing muscle movement

3. Transcranial magnetic stimulation (TMS)

Application - Cortical stimulation, many clinical and research applications
Patient state - Awake
Mechanism - Strong magnetic field (~1T) creates current in cortex. Suprathreshold stimulation causing action potentials

4. Transcranial direct current stimulation (tDCS)

Application - Cortical stimulation, mostly research applications
Patient state - Awake
Mechanism - Subthreshold stimulation which does not initiate action potentials. Causes a static hyper or depolarization of membrane potential. The mechanism is not fully understood but may have plasticity effects.

5. Standard (2 mA) transcranial alternating current stimulation (tACS)

Application - Cortical stimulation, research applications
Patient state - Awake
Mechanism - Subthreshold stimulation which does not initiate action potentials. Modulates membrane potential increasing or decreasing excitability.

Comparison of the Stimulation Strength of Transcranial Brain Stimulation Methods

Here we present a table comparing all the stimulation strength metrics for each of the five different transcranial brain stimulation methods. The values to calculate the metric are taken from the standard applications of these methods as reported in the literature[1,3–6]. Since TMS uses a magnetic field to induce a current within the brain, the only comparable metrics are electric field and current density within the brain as estimated based on computational models. tDCS uses direct current so the charge per phase or current density per phase cannot be calculated. MEP is often applied using cork-screw electrodes inserted into the skin, meaning that it is difficult to obtain an accurate estimate of electrode surface area. Therefore, current and change density metrics were not calculated. For MEP, modeling data estimating the current density or electric field strength in the brain was not available, nor was data on the current density in the brain for ECT or TMS.

The final column shows the metrics for 10 mA focused tACS applied for a duration of 2 minutes at 10 Hz. The MATLAB code containing all the values and formulae used to calculate the values in table 1 is included at the end of this report.

Table 1. Comparison of stimulation strength for different transcranial brain stimulation methods

	ECT[1]	MEP[3]	TMS[4]	tDCS[5]	Standard tACS [6]	Focused-tACS
**Current amplitude (mA)**	**800**	**100**	**N/A**	**2**	**2**	**10**
Phase duration (ms)	0.15	0.2	N/A	N/A	50	50
Charge per phase (mC)	0.12	0.02	N/A	N/A	0.06	0.3
Dose duration (s)	8	0.014		1200	1200	120
**Charge per dose (mC)**	**25**	**0.28**	**N/A**	**2400**	**1500**	**760**
Current density (mA/cm^2)	40	N/A	N/A	0.78	0.78	3.9
Charge density per phase (mC/cm^2)	0.006	N/A	N/A	N/A	0.0249	0.124
Charge density per dose (mC/cm^2)	1.27	N/A	N/A	937	597	298
Electric field (V/m)	100-200	N/A	100-200	1	1	3
Current density (A/m^2)	N/A	N/A	N/A	0.27	0.27	0.8

Risk Assessment of 10 mA Focused tACS

Based on these values presented in table 1 we assess the possibility of each of the theoretical risks listed at the beginning of the report could occur when 10 mA focused tACS is applied:

- **Damage to neural tissue - Not possible.**
  All reviewed methods are considered safe for research or clinical patient uses. None of the methods are known to damage neural tissue. Focused-tACS is well within the limits of the reviewed methods thus is not likely to damage neural tissue.

  It is important to note that a study by Leibetanz et al [7] examined the effect tDCS in rats on damage to neural tissue. Stimulation was applied directly to the skull of the rat – i.e. with the skin removed. They found that neural tissue damage began to occur at with a current density (as measured at the electrode surface) of 14.29 mA/cm^2 for tDCS This is below the value of 3.9 mA/cm^2 reported for focused tACS in the table above. However, it is important to point out that in humans the current must first pass through the skin and then a comparatively thick skull before reaching the brain. Both these factors mean that when tDCS or tACS is applied in humans, the current density value (as measured at the electrode surface) at which damage to neural tissue will occur will be significantly higher than 14.29 mA/cm^2.

- **Seizure - Very low.**
  ECT requires amplitudes of 800mA to cause seizure. Focused-tACS will be 80 times lower. Focused-tACS charge per dose is higher than ECT, but it is spread over a much longer time. Charge per does for 2 mins of focused-tACS charge is lower than for 20 min tDCS.

- **Skin burn - Very low.**
  None of the reviewed methods cause skin burns. Focused-tACS is well within the limits of the reviewed methods and thus will not cause skin burns. For any of the methods (with the exception of TMS), if the electrodes are not correctly attached to the skin the effective surface area decreases, thus increasing current and charge density. In the event of this occurring there is a theoretical possibility of skin burns for any of the reviewed methods.

- **Skin irritation - Low.**
  Isolated cases of skin irritation (temporary redness) have been reported with tDCS and standard tACS. Focused tACS will have a higher charge density per phase than standard tACS. Therefore, there may be an increased chance of skin irritation.

- **Pain (caused by stimulation of nerves in the scalp) – Likely, but mediated by local anesthetic.**
  Standard tACS and tDCS cause mild stimulation of touch and pain nerves in the scalp causing a tingling sensation. It is likely that the increased amplitude of focused-tACS will stimulate nerves in the skin directly under the electrode. Therefore, the use of a local anesthetic EMLA cream is recommended.

- **Tissue Heating – Low**.
  Tissue heating is dependent of current density, stimulation duration and a number of tissue properties. Our calculations show that current density is higher than all stimulation modalities except ECT. Given that ECT applies only extremely short pulses of energy, it can rely on thermal dissipation between pulses to minimize heating. tACS will be applied for longer periods of time. Thus, there is a possibility that tissue heating may occur. Therefore, it is recommend that initial experiments are conducted with lower stimulation levels, for shorter time periods, without anesthesia to allow the subject and experimenter to check for signs of tissue heating.

Stimulation amplitude and duration can then be gradually increased in a series of pilot experiments.

We conclude that the use of 10 mA focused tACS is likely to be safe. However, the increased current amplitude is likely to activate touch and pain nerves in the skin. Therefore, the use of a local anesthetic cream such as EMLA under the electrode is recommended. Since the current amplitude is higher there is also an increased risk of mild skin irritation such as redness. Therefore, it is recommend that the subject's skin is regularly checked for signs of irritation between periods of stimulation. Additionally, it is recommend that initial experiments are conducted at lower levels and without anesthesia to check for signs of possible tissue heating, before gradually building up to higher stimulation levels.

References

[1] Antal A, Paulus W. Transcranial alternating current stimulation (tACS). Front Hum Neurosci 2013;7:317. doi:10.3389/fnhum.2013.00317.

[2] McIntyre CC, Grill WM, Sherman DL, Thakor N V. Cellular effects of deep brain stimulation: model-based analysis of activation and inhibition. J Neurophysiol 2004;91:1457–69. doi:10.1152/jn.00989.2003.

[3] Peterchev a, Moacyr a, Deng Z-D, Lisanby S. ECT Stimulus Parameters: Rethinking dosage. J Ect 2011;26:159–74. doi:10.1097/YCT.0b013e3181e48165.ECT.

[4] Jameson LC. Monitoring the Nervous System for Anesthesiologists and Other Health Care Professionals. In: Koht A, Sloan TB, Toleikis JR, editors. J. Neurosurg. Anesthesiol., vol. 24, 2012, p. 226–7. doi:10.1097/ANA.0b013e31825622ae.

[5] Paffi A, Camera F, Carducci F, Rubino G, Tampieri P, Liberti M, et al. A computational model for real-time calculation of electric field due to transcranial magnetic stimulation in clinics. Int J Antennas Propag 2015;2015:23–7. doi:10.1155/2015/976854.

[6] Fregni F, Nitsche M a., Loo CK, Brunoni a. R, Marangolo P, Leite J, et al. Regulatory Considerations for the Clinical and Research Use of Transcranial Direct Current Stimulation (tDCS): review and recommendations from an expert panel. Clin Res Regul Aff 2015;32:22–35. doi:10.3109/10601333.2015.980944.

MATLAB code used to calculate values in table 1

```
S = [];
n=0;

% ECT - 800 mA, 8 sec
n = n+1;
S(n).name = 'ECT';
S(n).amplitude = 800; % mA
S(n).pulsedur = 0.15e-3;
S(n).dur = 8;
S(n).frequency = 13;
S(n).npulses = S(n).dur*S(n).frequency;
S(n).chargeperphase = S(n).amplitude * S(n).pulsedur; % mC
S(n).chargeperdose = S(n).amplitude * S(n).pulsedur*2 * S(n).npulses; % mC
S(n).electrodesurfacearea = pi*(5/2)^2; % cm2
S(n).currentdensity = S(n).amplitude/S(n).electrodesurfacearea; % mA/cm2
S(n).chargedensityperphase = S(n).chargeperphase/S(n).electrodesurfacearea; % mC/cm2
S(n).chargedensityperdose = S(n).chargeperdose/S(n).electrodesurfacearea; % mC/cm2

% MEP - 100 mA, 7 pulses
n = n+1;
S(n).name = 'MEP';
S(n).amplitude = 100;
S(n).pulsedur = 0.2e-3;
S(n).npulses = 7;
S(n).dur = 14e-3;
S(n).chargeperphase = S(n).amplitude * S(n).pulsedur;
S(n).chargeperdose = S(n).amplitude * S(n).pulsedur*2 * S(n).npulses;

% tDCS - 2mA, 20 min
n=n+1;
S(n).name = 'tDCS';
S(n).amplitude = 2;
S(n).dur = 20*60;
S(n).chargeperdose = S(n).amplitude*S(n).dur;
S(n).chargeperphase = NaN;
S(n).electrodesurfacearea = 1.6^2; % cm2
S(n).currentdensity = S(n).amplitude/S(n).electrodesurfacearea; % mA/cm2
S(n).chargedensityperphase = S(n).chargeperphase/S(n).electrodesurfacearea; % mC/cm2
S(n).chargedensityperdose = S(n).chargeperdose/S(n).electrodesurfacearea; % mC/cm2

% Standard tACS - 2mA, 20 min
n=n+1;
S(n).name = 'Standard tACS';
S(n).amplitude = 2;
frange = 10;
fs = 1000;
```

```
S(n).dur = 20*60;
tvec = [1/fs:1/fs:S(n).dur];
s = S(n).amplitude*sin(2*pi*tvec*frange);
S(n).chargeperdose = sum(abs(s))*(1/fs);
ind = round((1/frange)/2*1000);
S(n).chargeperphase = sum(abs(s(1:ind)))*(1/fs);
S(n).pulsedur = tvec(ind);
S(n).electrodesurfacearea = 1.6^2; % cm2
S(n).currentdensity = S(n).amplitude/S(n).electrodesurfacearea; % mA/cm2
S(n).chargedensityperphase = S(n).chargeperphase/S(n).electrodesurfacearea; % mC/cm2
S(n).chargedensityperdose = S(n).chargeperdose/S(n).electrodesurfacearea; % mC/cm2

% Focused tACS - 10mA, 2 min
n=n+1;
S(n).name = 'Focused-TACS';
S(n).amplitude = 10;
frange = 10;
fs = 1000;
S(n).dur = 2*60;
tvec = [1/fs:1/fs:S(n).dur];
s = S(n).amplitude*sin(2*pi*tvec*frange);
S(n).chargeperdose = sum(abs(s))*(1/fs);
ind = round((1/frange)/2*1000);
S(n).chargeperphase = sum(abs(s(1:ind)))*(1/fs);
S(n).pulsedur = tvec(ind);
S(n).electrodesurfacearea = 1.6^2; % cm2
S(n).currentdensity = S(n).amplitude/S(n).electrodesurfacearea; % mA/cm2
S(n).chargedensityperphase = S(n).chargeperphase/S(n).electrodesurfacearea; % mC/cm2
S(n).chargedensityperdose = S(n).chargeperdose/S(n).electrodesurfacearea; % mC/cm2
```

Using high-amplitude and focused transcranial alternating current stimulation to entrain physiological tremor

Authors

Ahmad Khatoun, Jolien Breukers, Sara Op de Beeck, Ioana Gabriela Nica, Jean-Marie Aerts, Laura Seynaeve, Tom Haeck, Boateng Asamoah, Myles Mc Laughlin

Supplementary Results

Individual Phase Entrainment Exp 1: Histograms

Phase-difference probability histograms calculated for all subjects and for all sessions in Experiment 1 for the conditions (OFF, MC and PFC). X-axis represents the phase difference and the y-axis represents the occurrence probability. Each row represent different session number (refer to Fig.2 in the manuscript for more information).

Subject 1.1

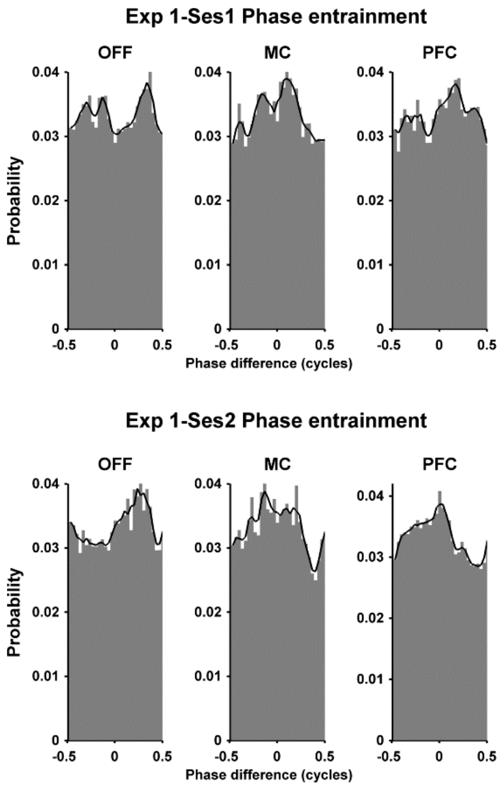

Subject 1.2

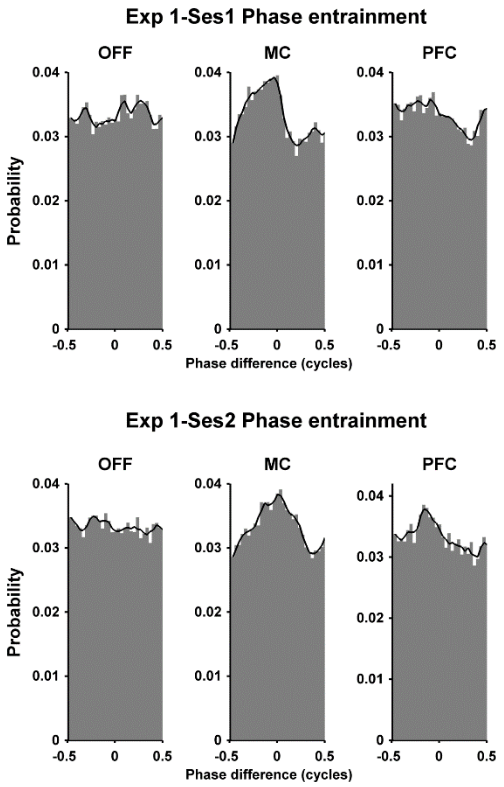

Subject 1.3

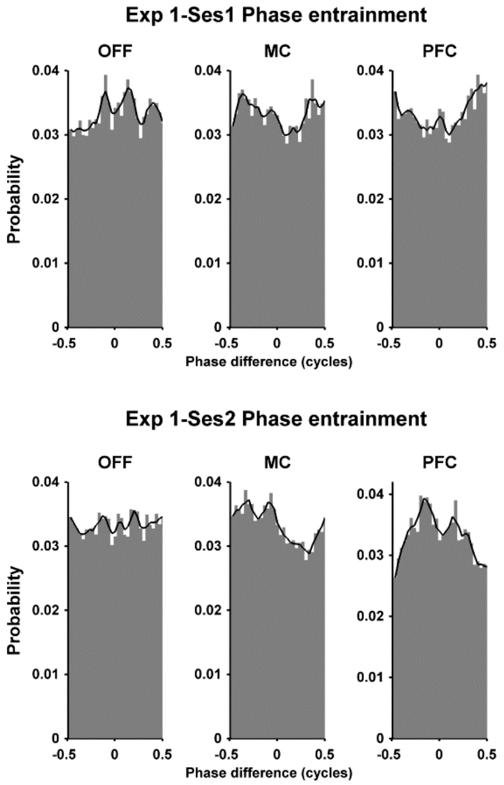

Subject 1.4

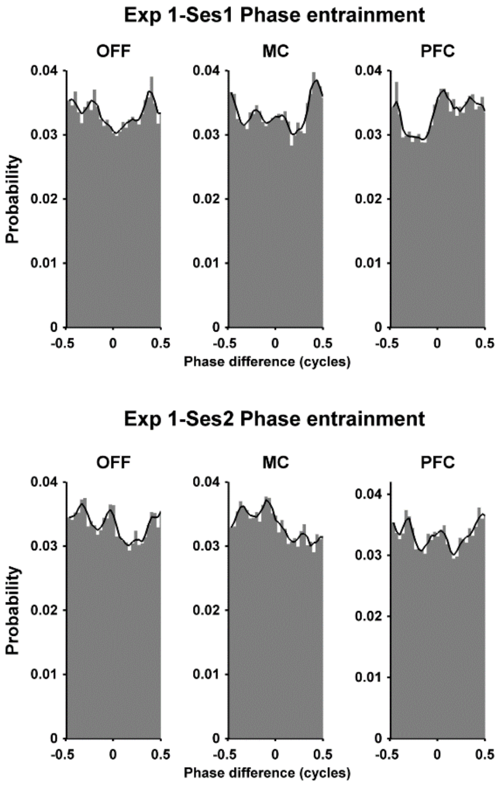

Subject 1.5

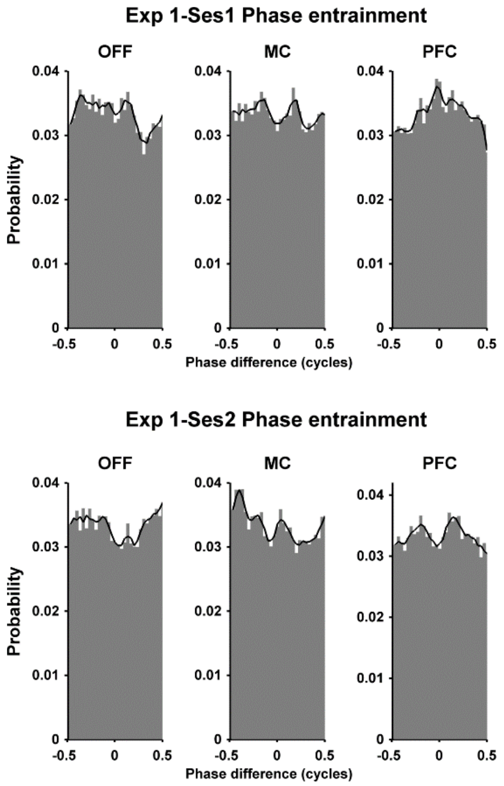

Subject 1.6

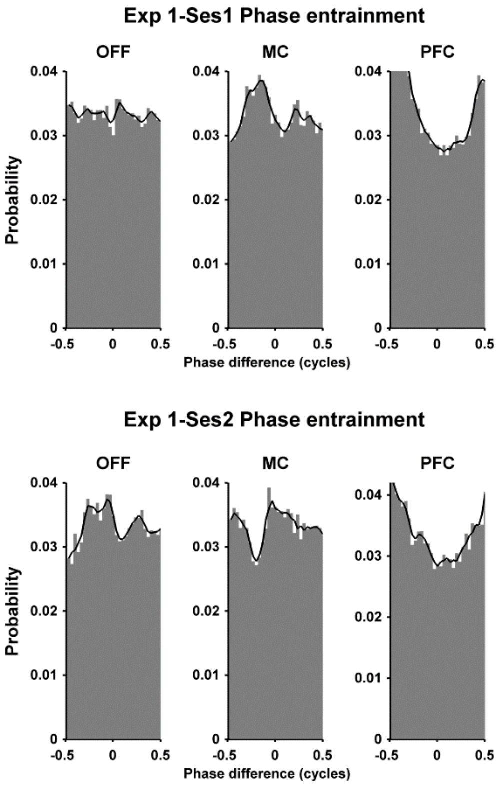

Subject 1.7

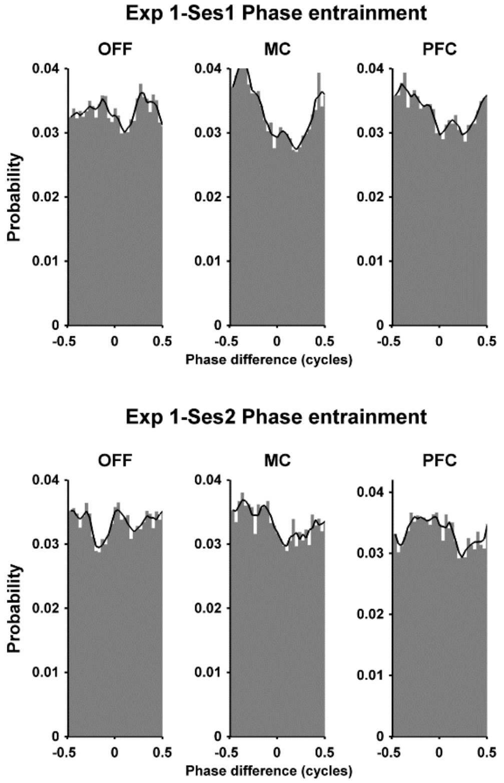

Subject 1.8

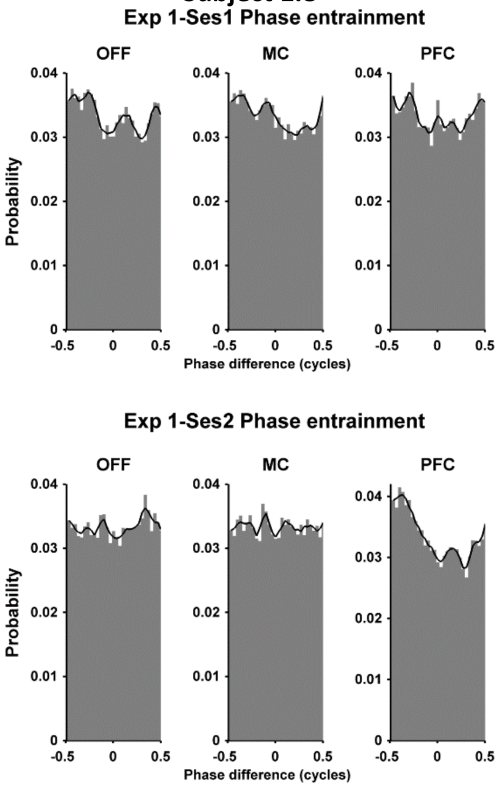

Subject 1.9

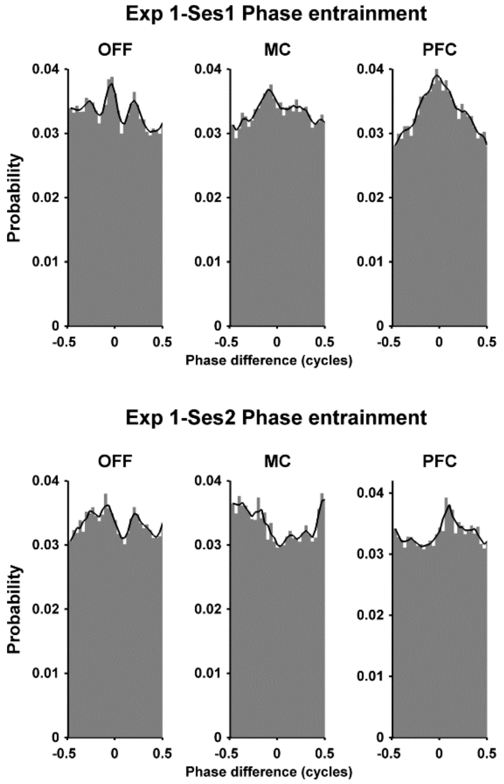

Subject 1.10

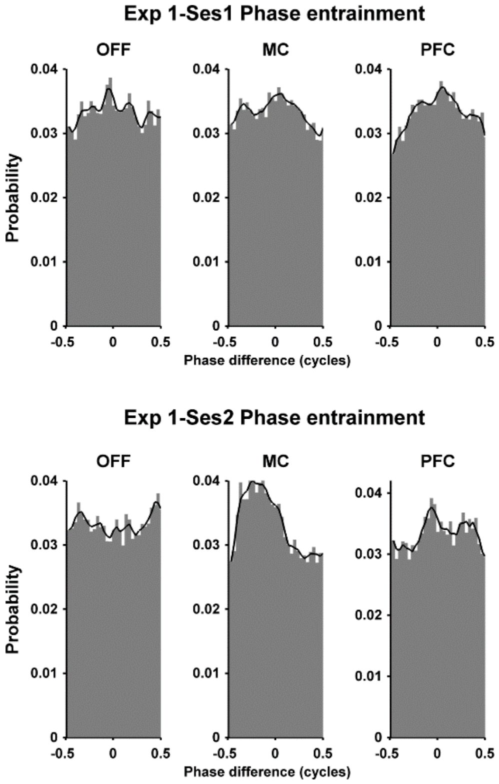

Individual Phase Entrainment Exp 2: Histograms

Phase-difference probability histograms calculated for all subjects and for all sessions in Experiment 2 for the conditions (OFF, MC and OC). X-axis represents the phase difference and the y-axis represents the occurrence probability. Each row represent different session number (refer to Fig.2 in the manuscript for more information).

Subject 2.1

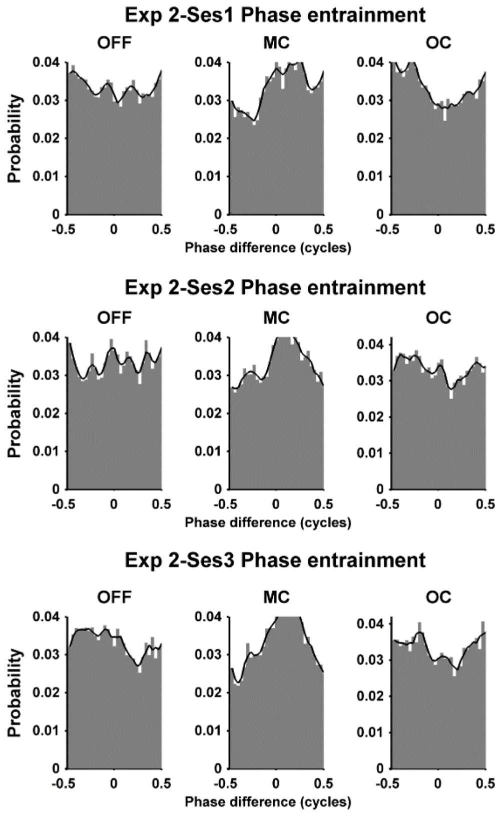

Subject 2.2

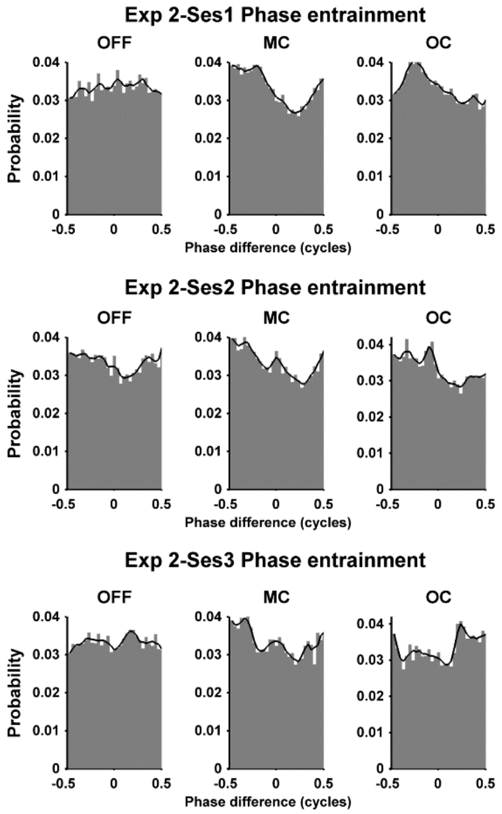

Subject 2.3

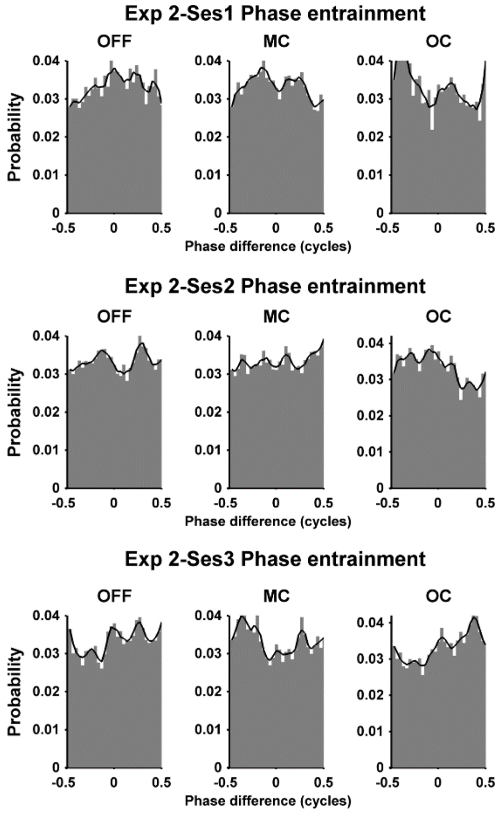

Subject 2.4

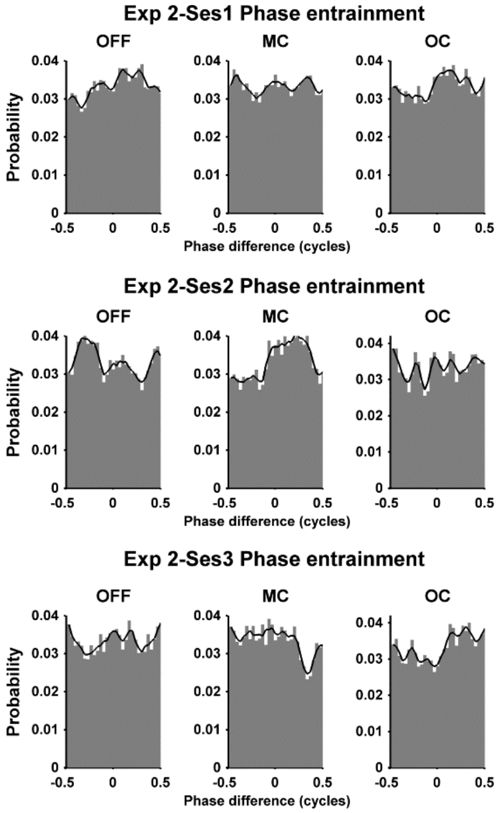

Subject 2.11

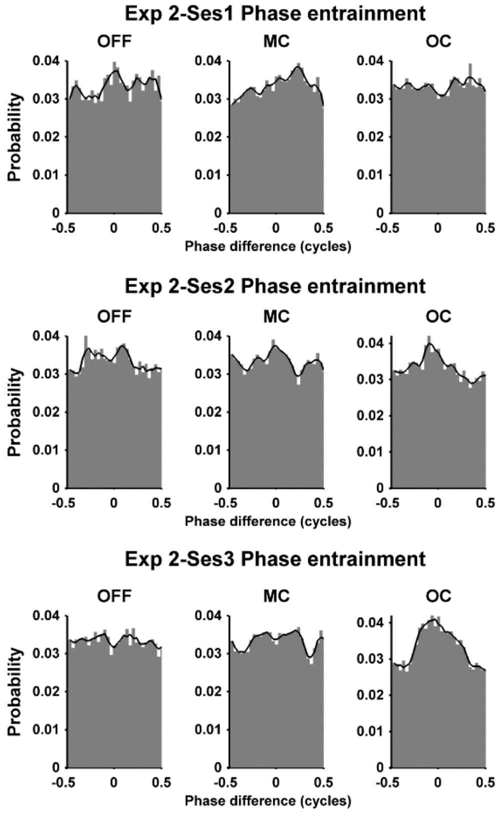

Subject 2.12

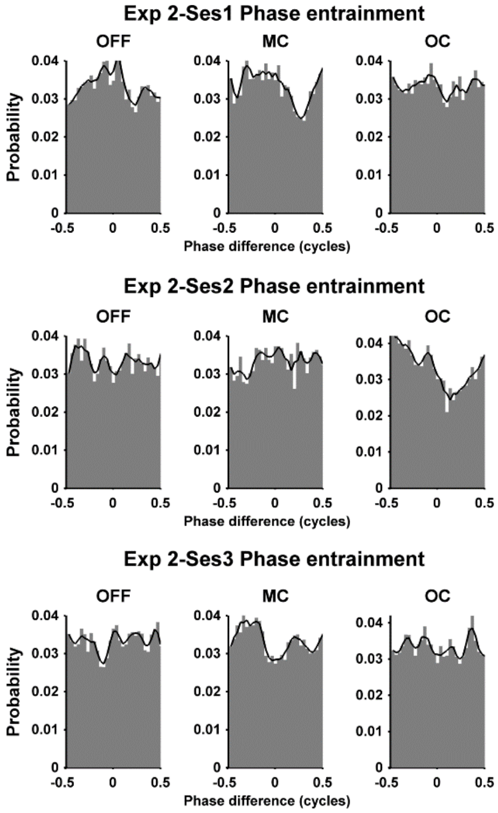

Subject 2.13

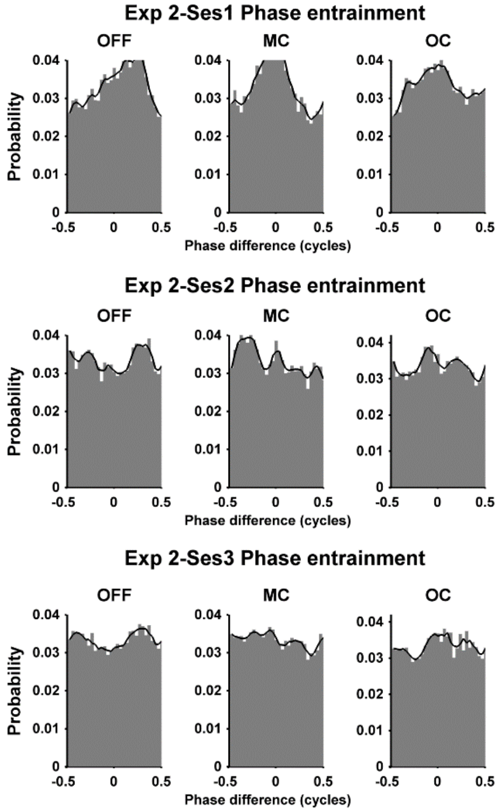

Subject 2.14

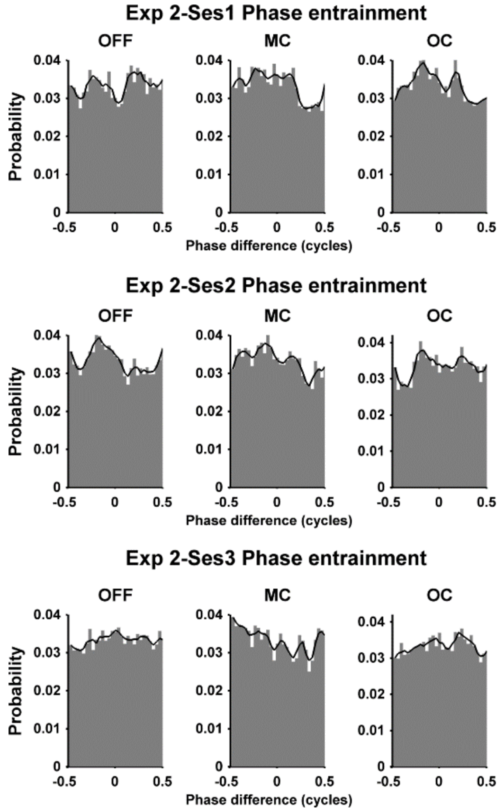

Subject 2.15

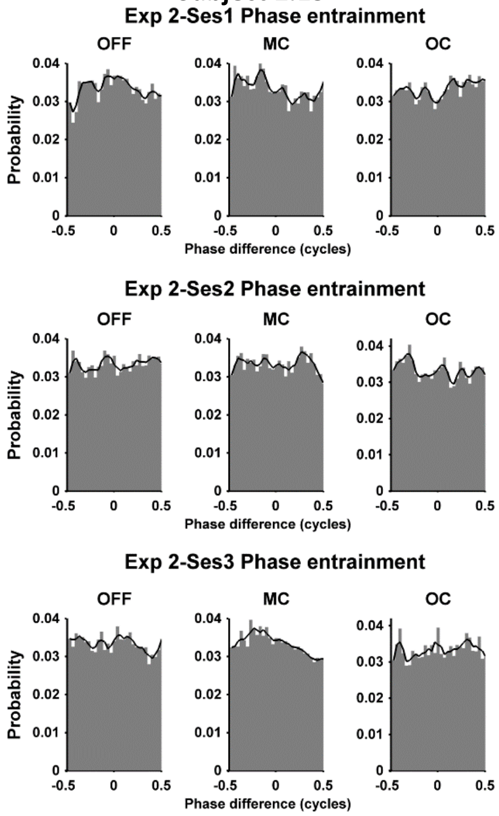

Subject 2.16

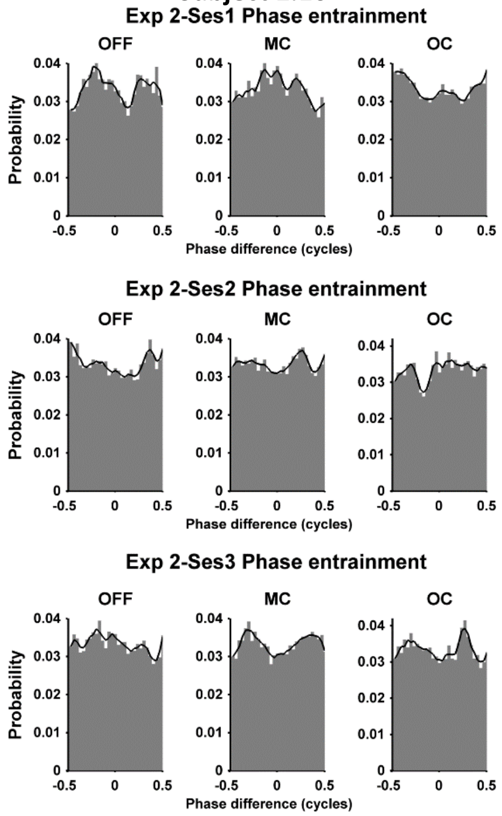

Individual Phase Entrainment Exp 1: Averaged Data

The phase entrainment PLV calculated for all subjects and conditions in Experiment 1. X-axis represents the condition and y-axis the PLV value. Different sessions are denoted by different colors and the average for all sessions is shown in gray.

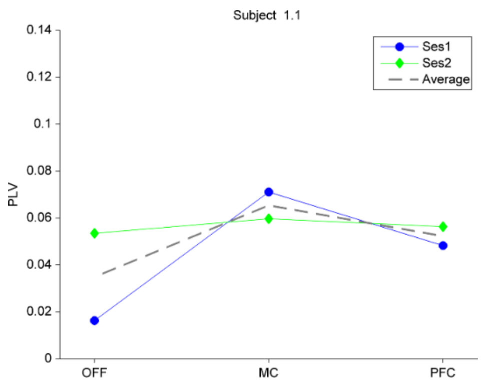

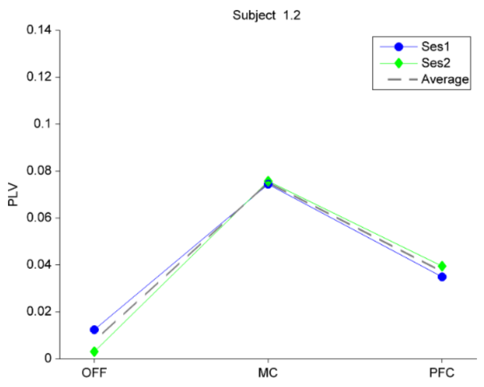

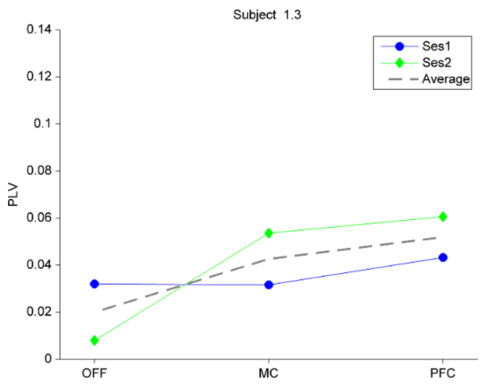

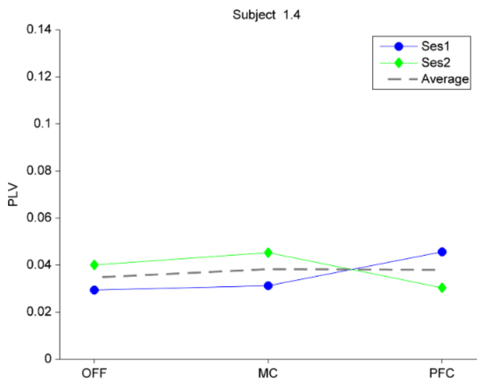

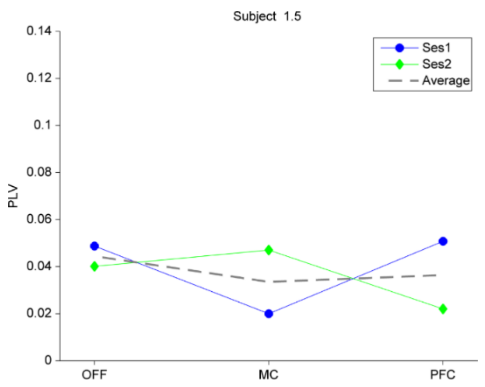

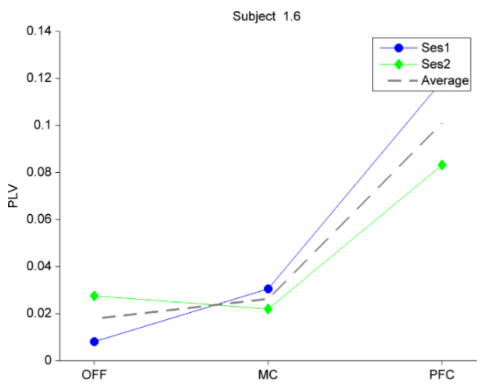

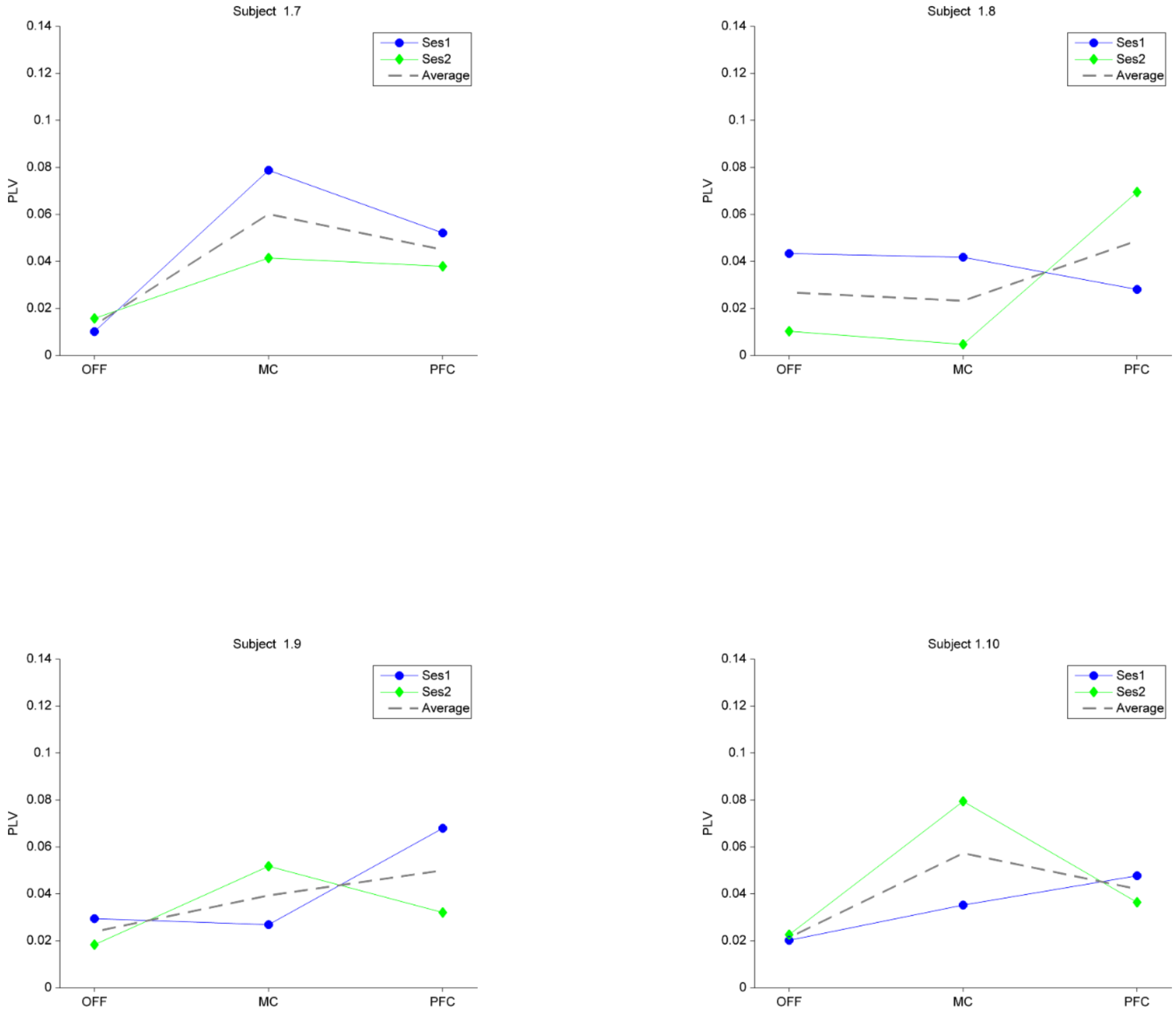
Subject 1.7
Subject 1.8
Subject 1.9
Subject 1.10
PLV
0.14
0.12
0.1
0.08
0.06
0.04
0.02
0
OFF
MC
PFC
Ses1
Ses2
Average

Individual Phase Entrainment Exp 2: Averaged Data

The phase entrainment PLV calculated for all subjects and conditions in Experiment 2. X-axis represents the condition and y-axis the PLV value. Different sessions are denoted by different colors and the average for all sessions is shown in gray.

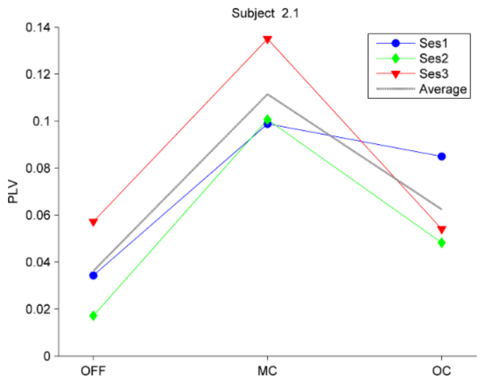

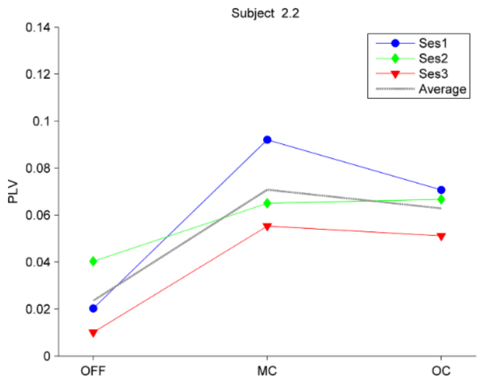

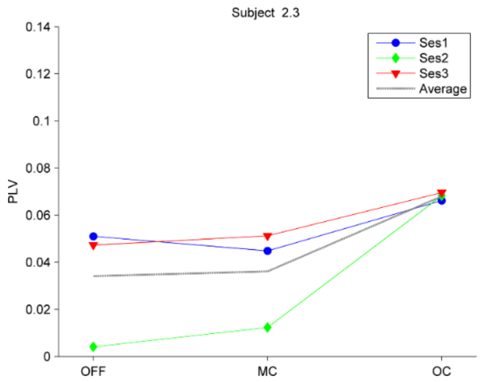

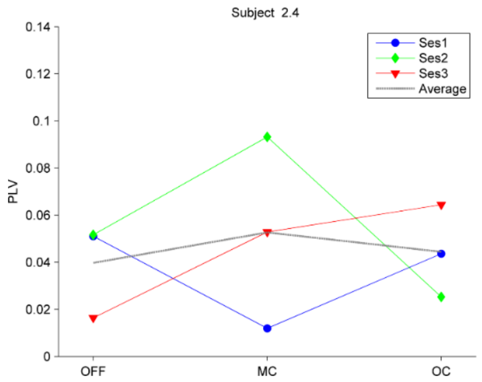

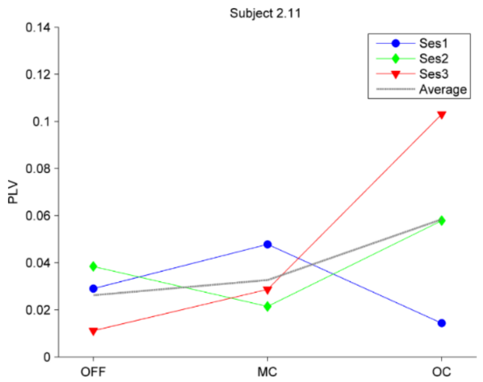

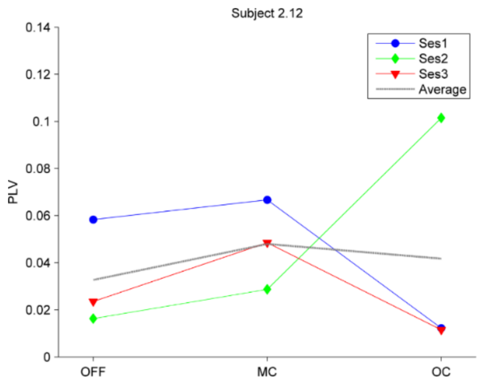

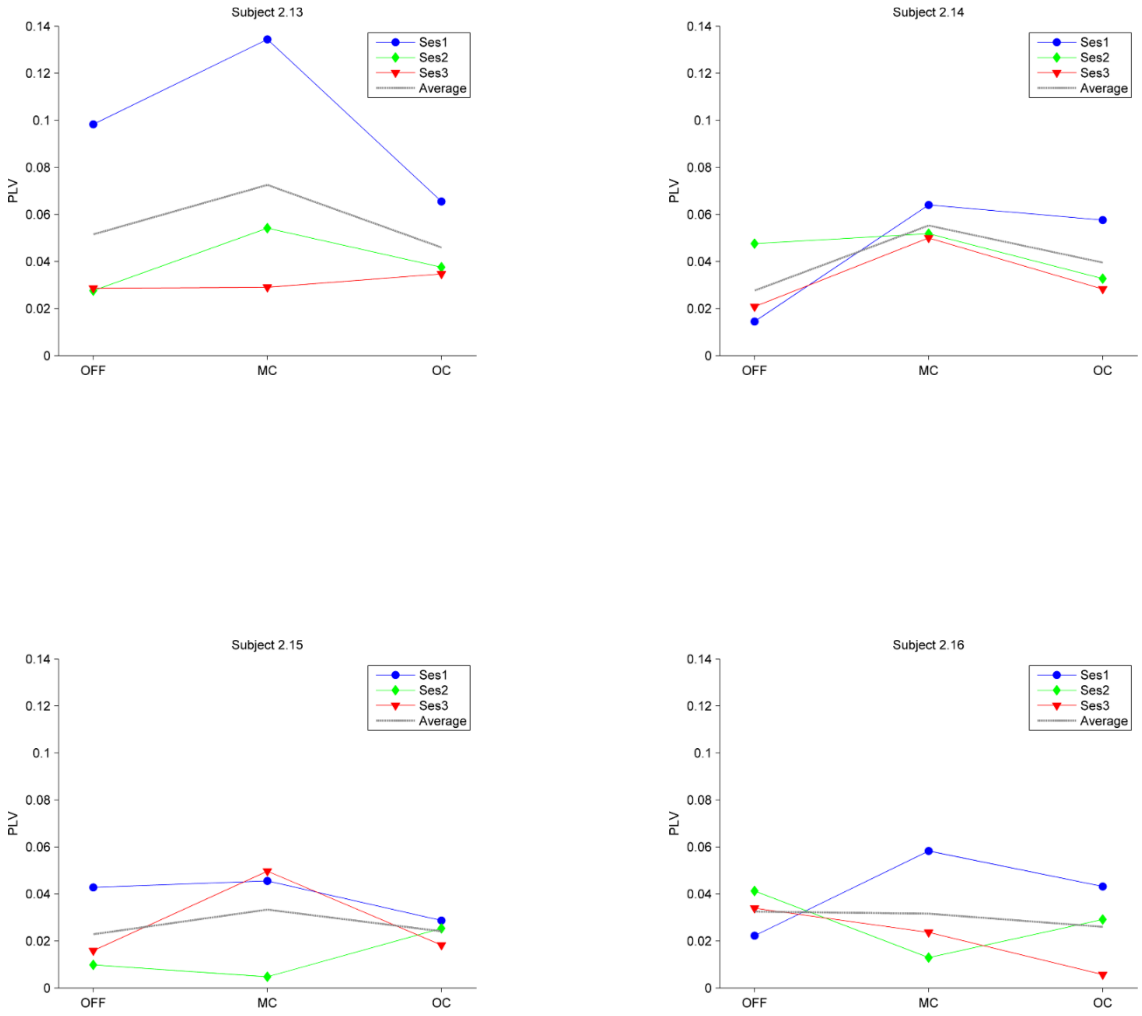
Subject 2.13
Subject 2.14
Subject 2.15
Subject 2.16
PLV
0
0.02
0.04
0.06
0.08
0.1
0.12
0.14
OFF
MC
OC
Ses1
Ses2
Ses3
Average

Individual Amplitude Modulation Exp1

Tremor amplitude modulation functions illustrating the effect (if any) of tACS on physiological tremor amplitude calculated for all subjects and conditions in Experiment 1. For each panel, the x-axis shows the phase-difference between tremor and tACS and the y-axis shows the average tremor amplitude that occurred at that phase-difference normalized across all phase-differences for that condition and then expressed as a percentage. This is similar to Fig.4 in the manuscript.

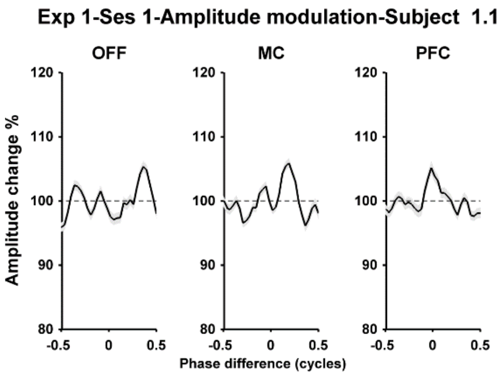

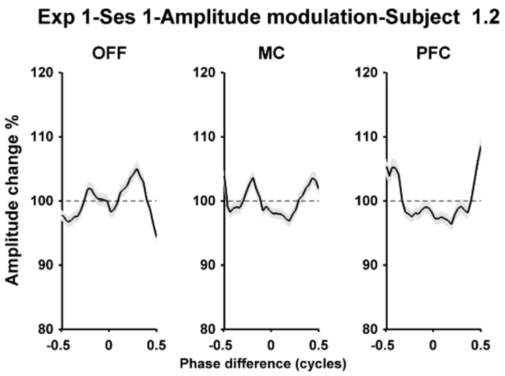

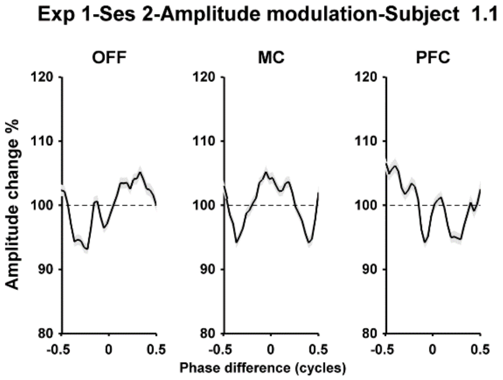

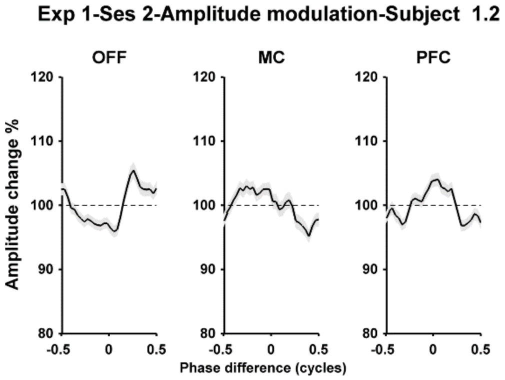

Exp 1-Ses 1-Amplitude modulation-Subject 1.3

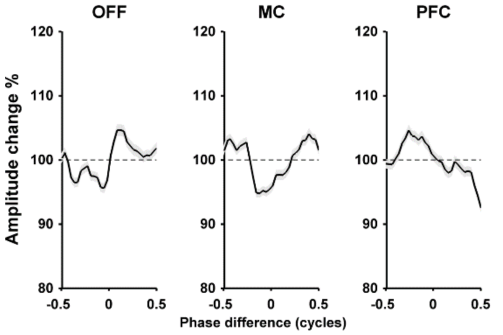

Exp 1-Ses 1-Amplitude modulation-Subject 1.4

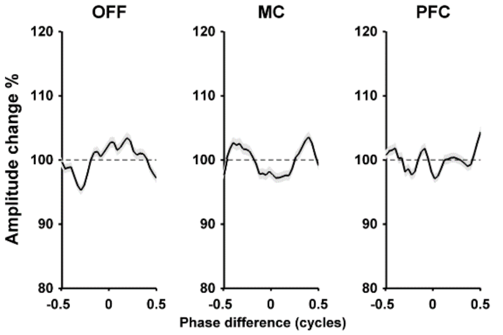

Exp 1-Ses 2-Amplitude modulation-Subject 1.3

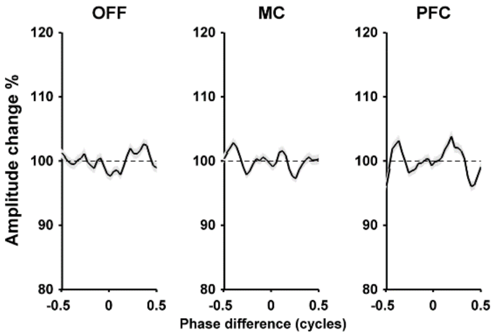

Exp 1-Ses 2-Amplitude modulation-Subject 1.4

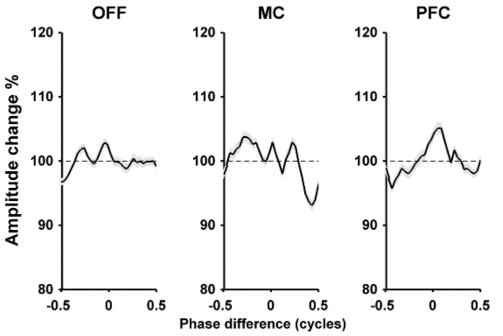

Exp 1-Ses 1-Amplitude modulation-Subject 1.5

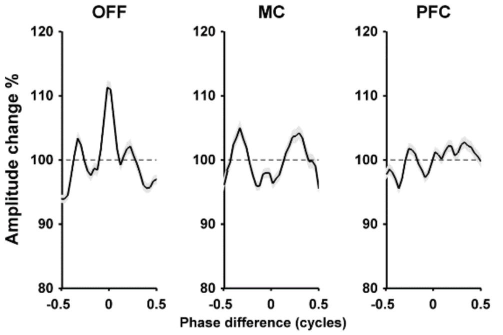

Exp 1-Ses 1-Amplitude modulation-Subject 1.6

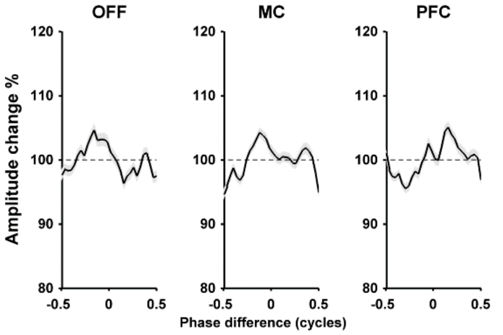

Exp 1-Ses 2-Amplitude modulation-Subject 1.5

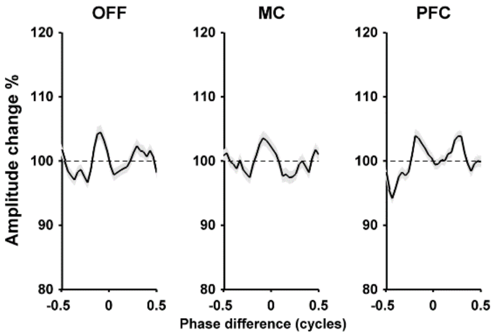

Exp 1-Ses 2-Amplitude modulation-Subject 1.6

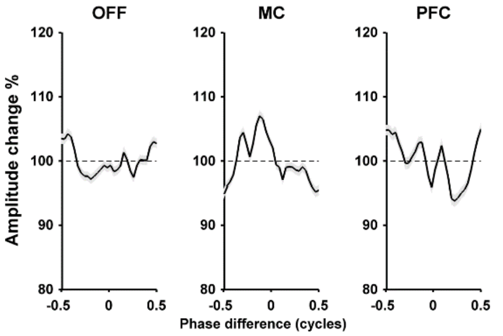

Exp 1-Ses 1-Amplitude modulation-Subject 1.7

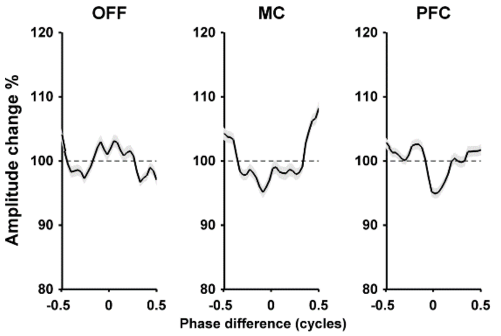

Exp 1-Ses 1-Amplitude modulation-Subject 1.8

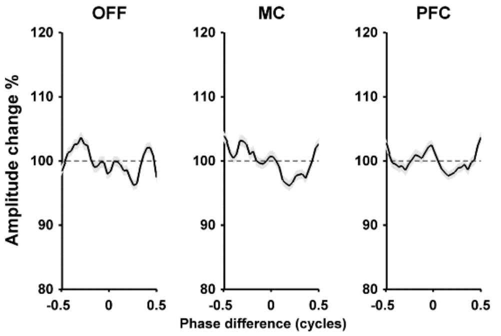

Exp 1-Ses 2-Amplitude modulation-Subject 1.7

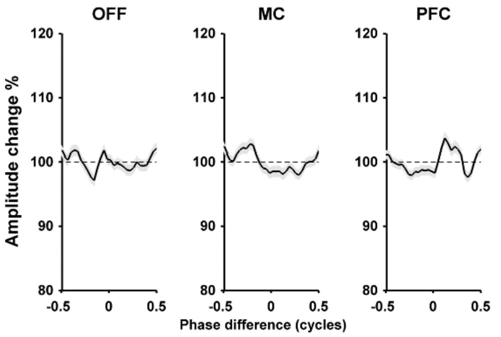

Exp 1-Ses 2-Amplitude modulation-Subject 1.8

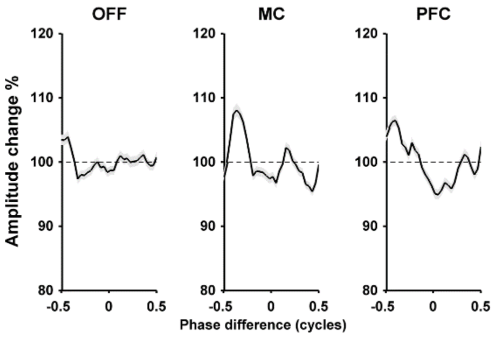

Exp 1-Ses 1-Amplitude modulation-Subject 1.9

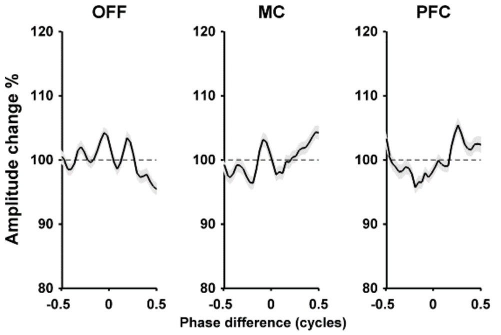

Exp 1-Ses 1-Amplitude modulation-Subject 1.10

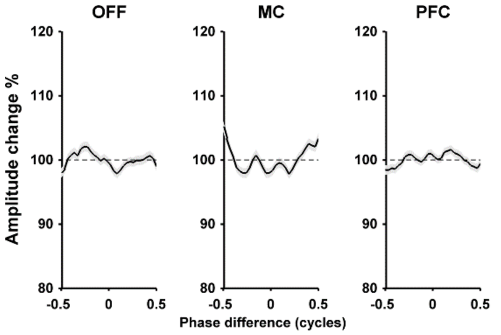

Exp 1-Ses 2-Amplitude modulation-Subject 1.9

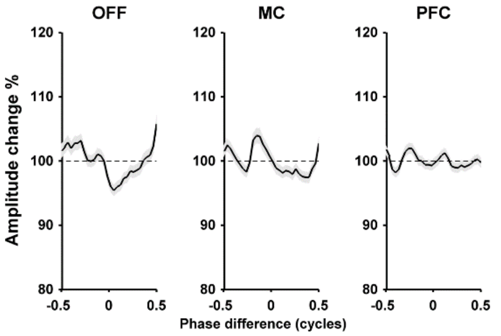

Exp 1-Ses 2-Amplitude modulation-Subject 1.10

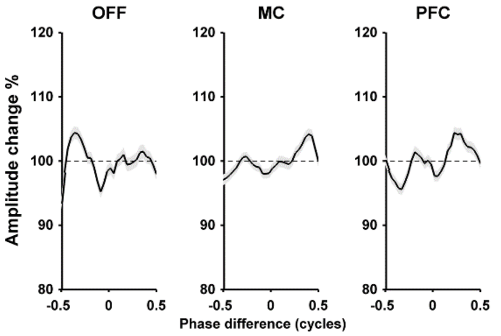

Individual Amplitude Modulation Exp2

Tremor amplitude modulation functions illustrating the effect (if any) of tACS on physiological tremor amplitude calculated for all subjects and conditions in Experiment 2. For each panel, the x-axis shows the phase-difference between tremor and tACS and the y-axis shows the average tremor amplitude that occurred at that phase-difference normalized across all phase-differences for that condition and then expressed as a percentage. This is similar to Fig.4 in the manuscript.

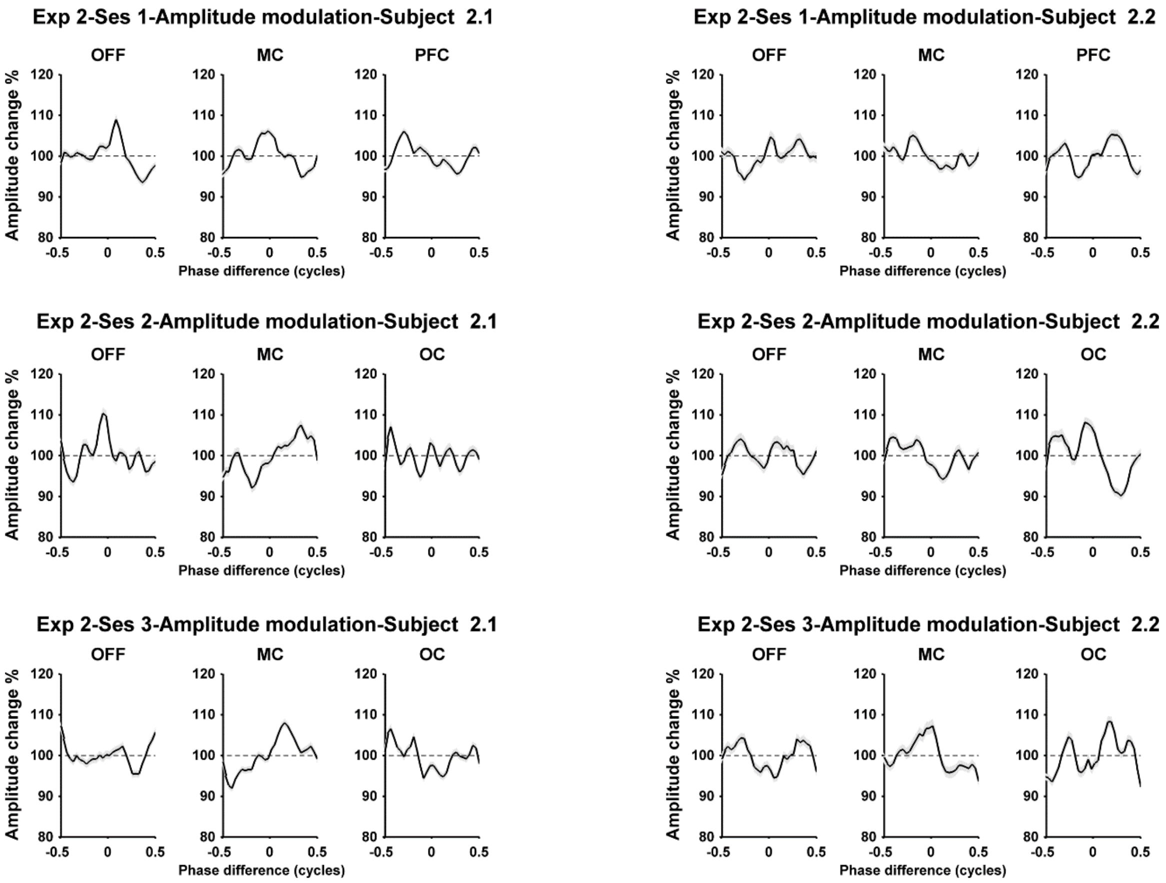

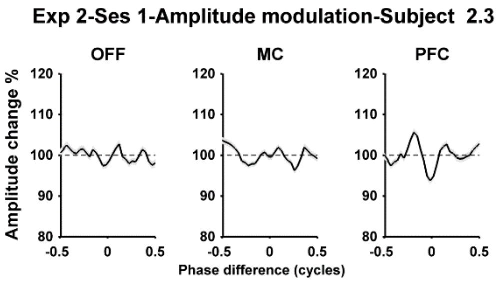
Exp 2-Ses 1-Amplitude modulation-Subject 2.3
OFF
MC
PFC
Amplitude change %
120
110
100
90
80
-0.5
0
0.5
Phase difference (cycles)

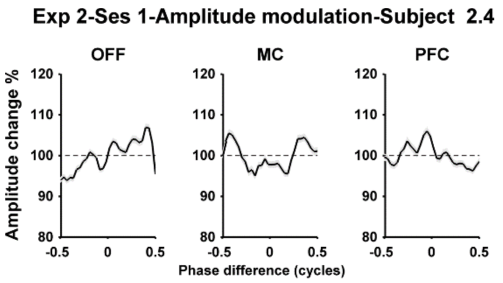
Exp 2-Ses 1-Amplitude modulation-Subject 2.4
OFF
MC
PFC
Amplitude change %
120
110
100
90
80
-0.5
0
0.5
Phase difference (cycles)

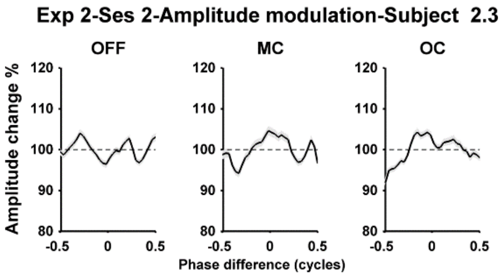
Exp 2-Ses 2-Amplitude modulation-Subject 2.3
OFF
MC
OC
Amplitude change %
120
110
100
90
80
-0.5
0
0.5
Phase difference (cycles)

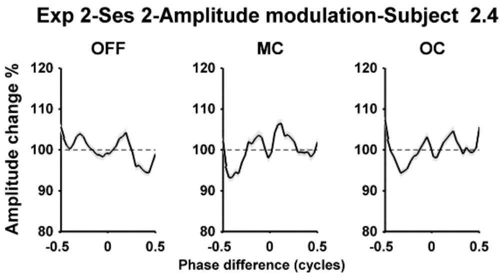
Exp 2-Ses 2-Amplitude modulation-Subject 2.4
OFF
MC
OC
Amplitude change %
120
110
100
90
80
-0.5
0
0.5
Phase difference (cycles)

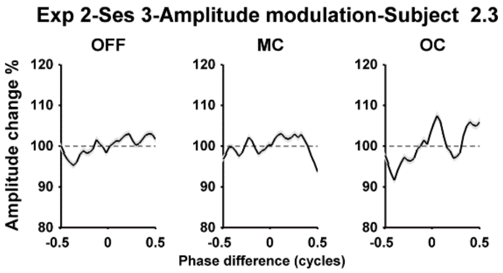
Exp 2-Ses 3-Amplitude modulation-Subject 2.3
OFF
MC
OC
Amplitude change %
120
110
100
90
80
-0.5
0
0.5
Phase difference (cycles)

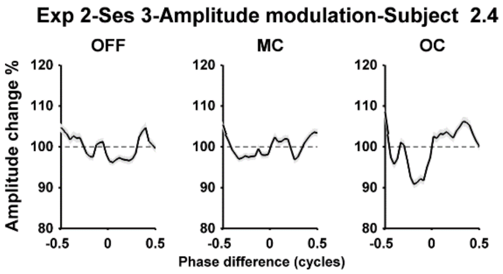
Exp 2-Ses 3-Amplitude modulation-Subject 2.4
OFF
MC
OC
Amplitude change %
120
110
100
90
80
-0.5
0
0.5
Phase difference (cycles)

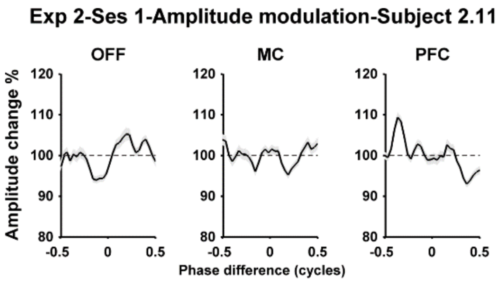
Exp 2-Ses 1-Amplitude modulation-Subject 2.11
OFF
MC
PFC
Amplitude change %
120
110
100
90
80
-0.5
0
0.5
Phase difference (cycles)

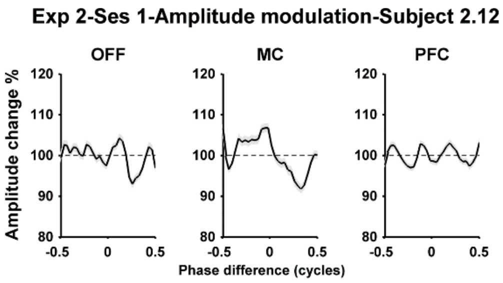
Exp 2-Ses 1-Amplitude modulation-Subject 2.12
OFF
MC
PFC
Amplitude change %
120
110
100
90
80
-0.5
0
0.5
Phase difference (cycles)

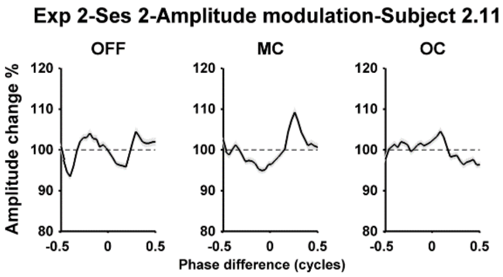
Exp 2-Ses 2-Amplitude modulation-Subject 2.11
OFF
MC
OC
Amplitude change %
120
110
100
90
80
-0.5
0
0.5
Phase difference (cycles)

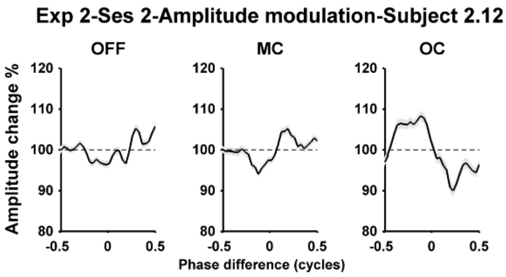
Exp 2-Ses 2-Amplitude modulation-Subject 2.12
OFF
MC
OC
Amplitude change %
120
110
100
90
80
-0.5
0
0.5
Phase difference (cycles)

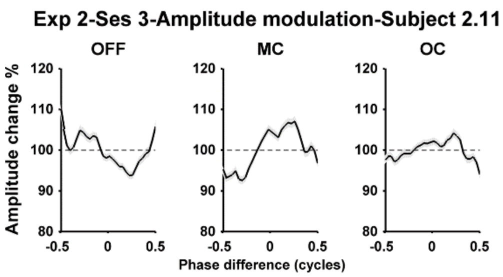
Exp 2-Ses 3-Amplitude modulation-Subject 2.11
OFF
MC
OC
Amplitude change %
120
110
100
90
80
-0.5
0
0.5
Phase difference (cycles)

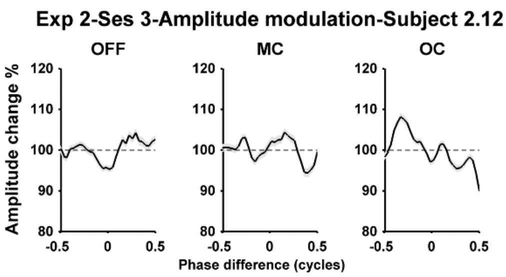
Exp 2-Ses 3-Amplitude modulation-Subject 2.12
OFF
MC
OC
Amplitude change %
120
110
100
90
80
-0.5
0
0.5
Phase difference (cycles)

Exp 2-Ses 1-Amplitude modulation-Subject 2.13

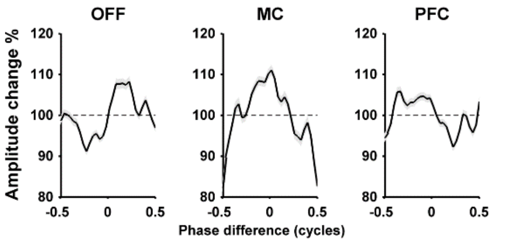

Exp 2-Ses 1-Amplitude modulation-Subject 2.14

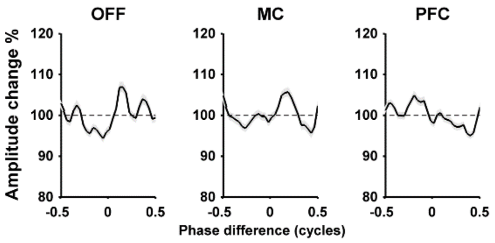

Exp 2-Ses 2-Amplitude modulation-Subject 2.13

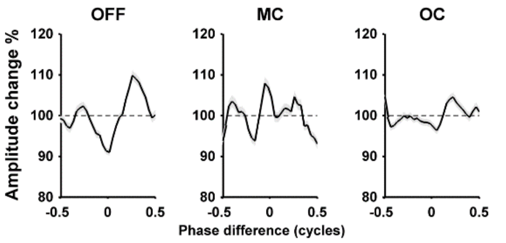

Exp 2-Ses 2-Amplitude modulation-Subject 2.14

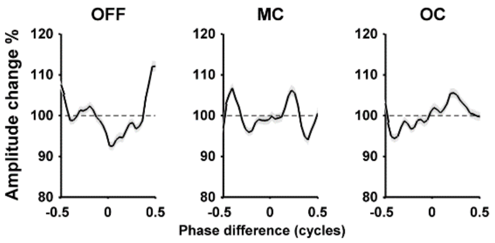

Exp 2-Ses 3-Amplitude modulation-Subject 2.13

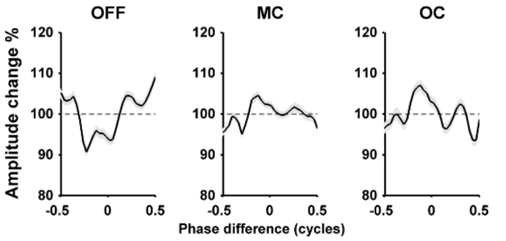

Exp 2-Ses 3-Amplitude modulation-Subject 2.14

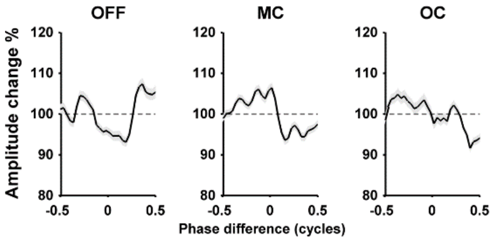

Exp 2-Ses 1-Amplitude modulation-Subject 2.15

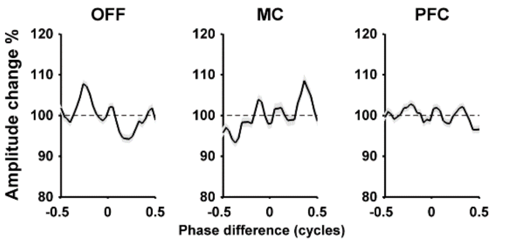

Exp 2-Ses 1-Amplitude modulation-Subject 2.16

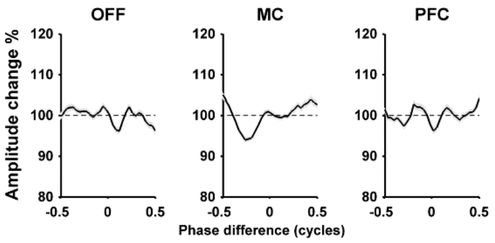

Exp 2-Ses 2-Amplitude modulation-Subject 2.15

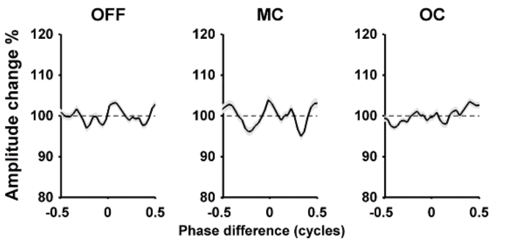

Exp 2-Ses 2-Amplitude modulation-Subject 2.16

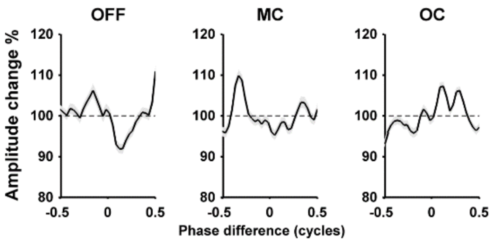

Exp 2-Ses 3-Amplitude modulation-Subject 2.15

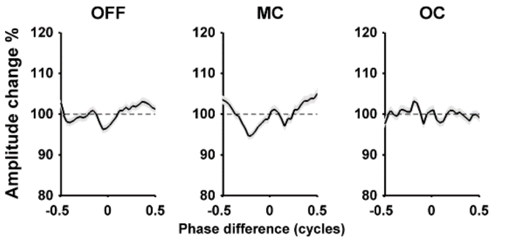

Exp 2-Ses 3-Amplitude modulation-Subject 2.16

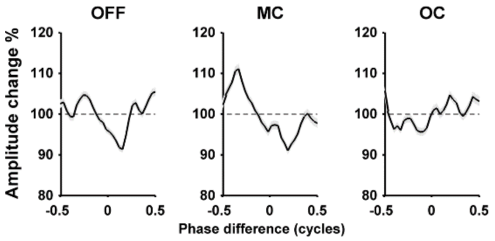

Individual Amplitude Modulation Exp1: Averaged Data

The amplitude modulation PLV calculated for all subjects and conditions in Experiment 1. X-axis represents the condition and y-axis the amplitude modulation PLV value. Different sessions are denoted by different colors and the average for all sessions is shown in gray.

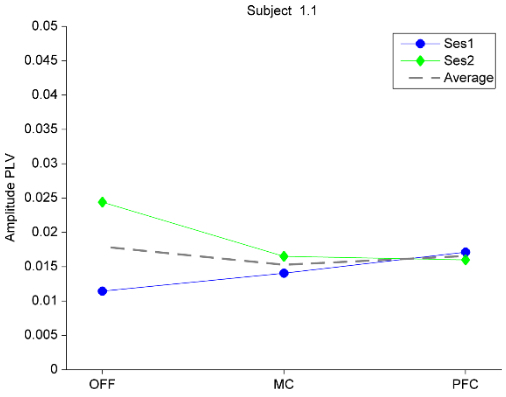

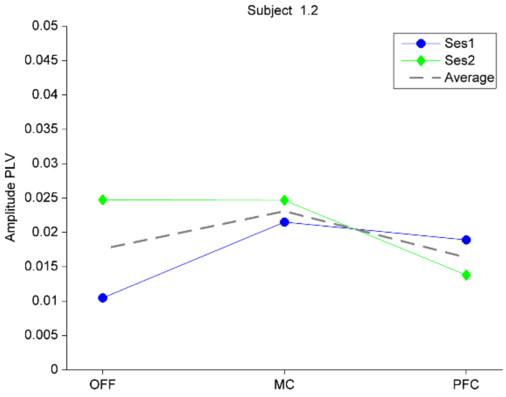

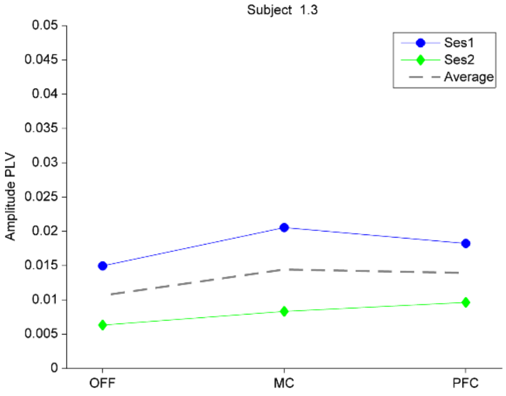

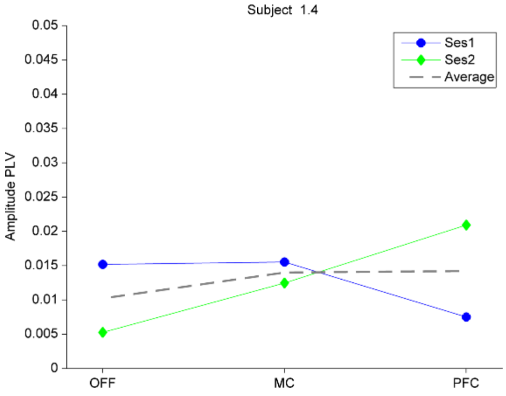

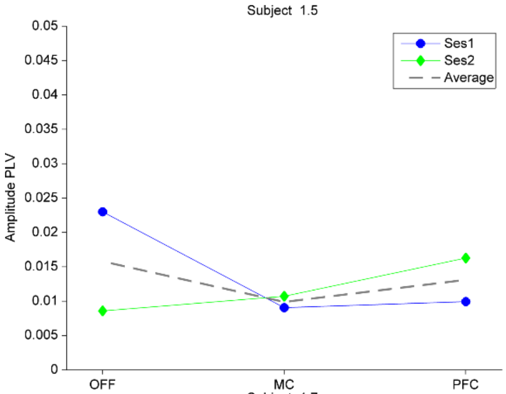
Subject 1.5
Amplitude PLV
0.05
0.045
0.04
0.035
0.03
0.025
0.02
0.015
0.01
0.005
0
OFF
MC
PFC
Ses1
Ses2
Average

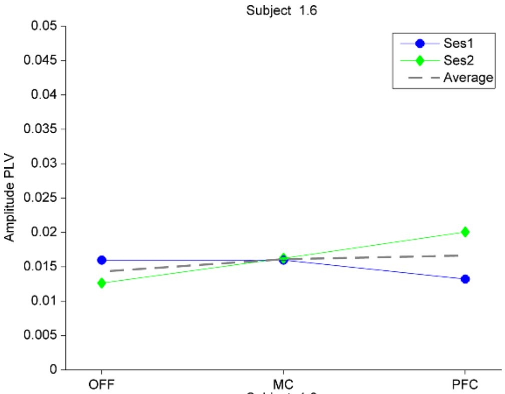
Subject 1.6
Amplitude PLV
0.05
0.045
0.04
0.035
0.03
0.025
0.02
0.015
0.01
0.005
0
OFF
MC
PFC
Ses1
Ses2
Average

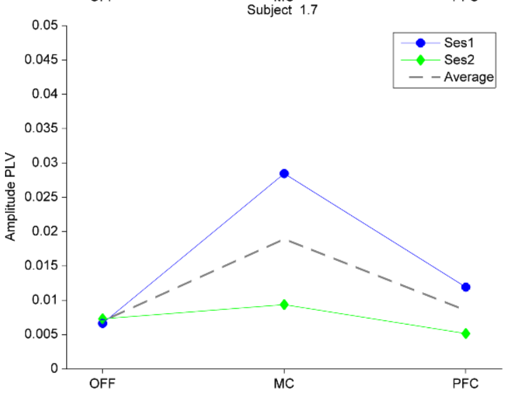
Subject 1.7
Amplitude PLV
0.05
0.045
0.04
0.035
0.03
0.025
0.02
0.015
0.01
0.005
0
OFF
MC
PFC
Ses1
Ses2
Average

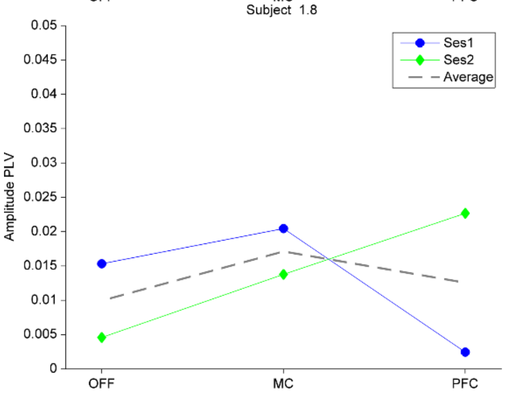
Subject 1.8
Amplitude PLV
0.05
0.045
0.04
0.035
0.03
0.025
0.02
0.015
0.01
0.005
0
OFF
MC
PFC
Ses1
Ses2
Average

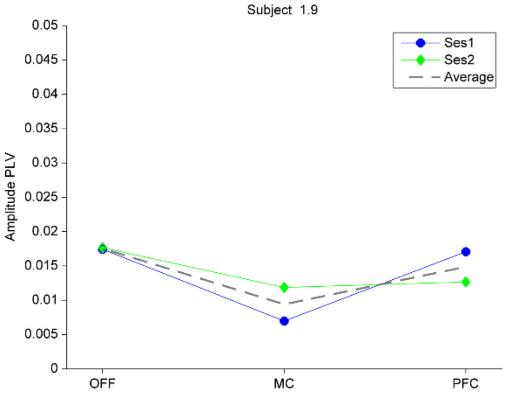
Subject 1.9
Amplitude PLV
0.05
0.045
0.04
0.035
0.03
0.025
0.02
0.015
0.01
0.005
0
OFF
MC
PFC
Ses1
Ses2
Average

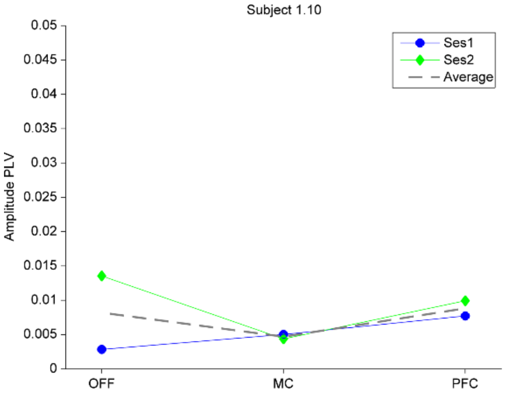
Subject 1.10
Amplitude PLV
0.05
0.045
0.04
0.035
0.03
0.025
0.02
0.015
0.01
0.005
0
OFF
MC
PFC
Ses1
Ses2
Average

Individual Amplitude Modulation Exp2: Averaged Data

The amplitude modulation PLV calculated for all subjects and conditions in Experiment 2. X-axis represents the condition and y-axis the amplitude modulation PLV value. Different sessions are denoted by different colors and the average for all sessions is shown in gray.

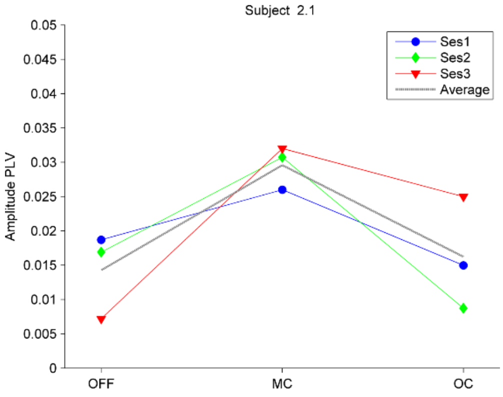

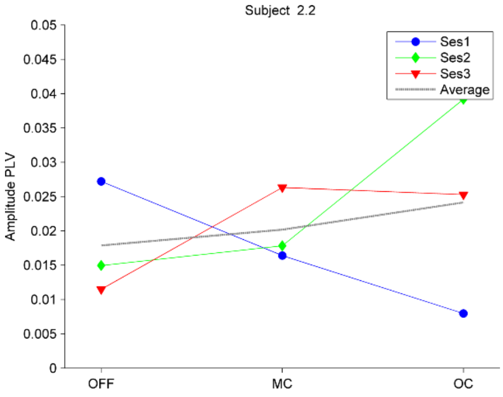

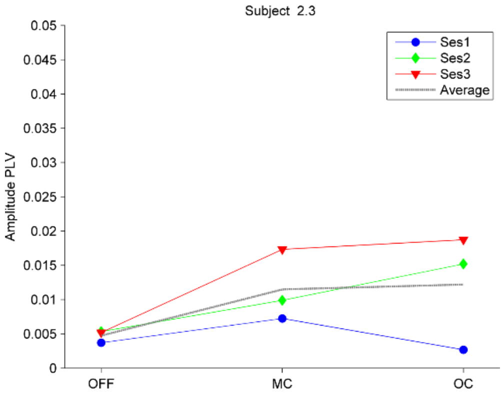

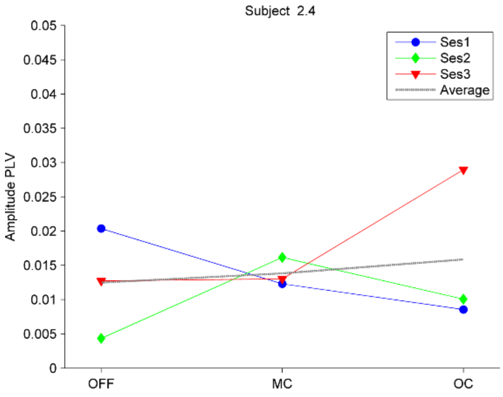

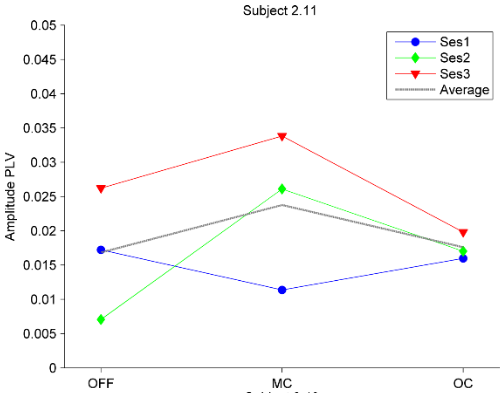
Subject 2.11
Amplitude PLV
Ses1
Ses2
Ses3
Average
OFF
MC
OC
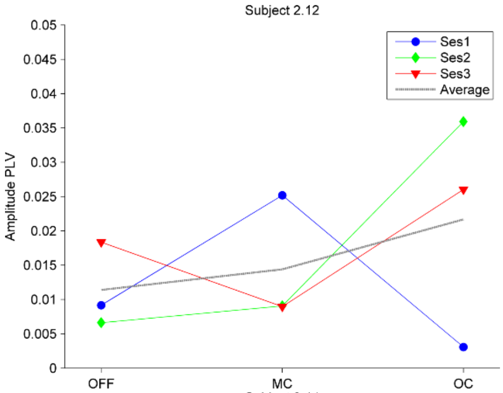
Subject 2.12
Amplitude PLV
Ses1
Ses2
Ses3
Average
OFF
MC
OC
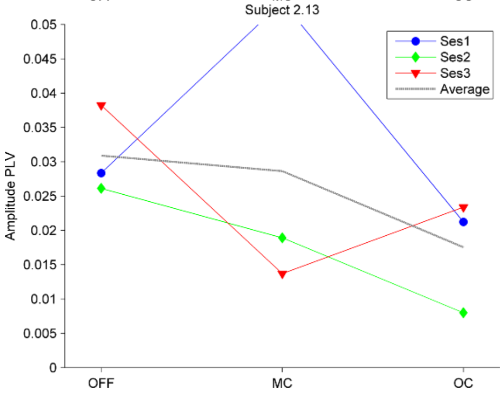
Subject 2.13
Amplitude PLV
Ses1
Ses2
Ses3
Average
OFF
MC
OC
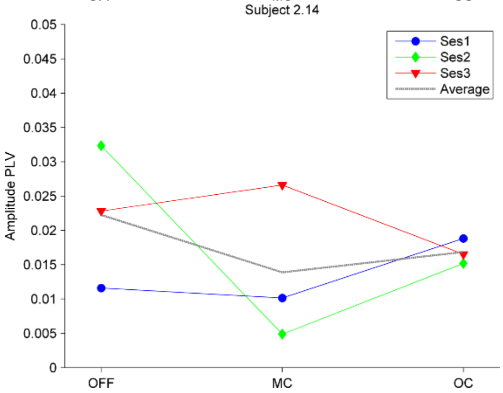
Subject 2.14
Amplitude PLV
Ses1
Ses2
Ses3
Average
OFF
MC
OC
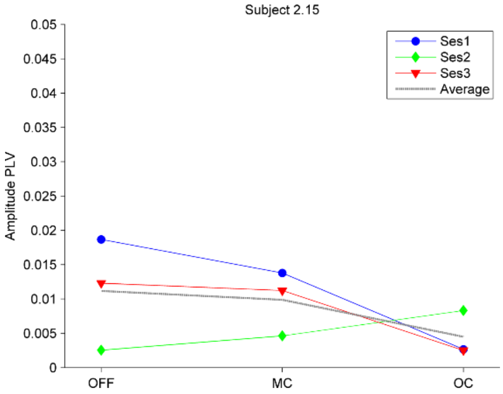
Subject 2.15
Amplitude PLV
Ses1
Ses2
Ses3
Average
OFF
MC
OC
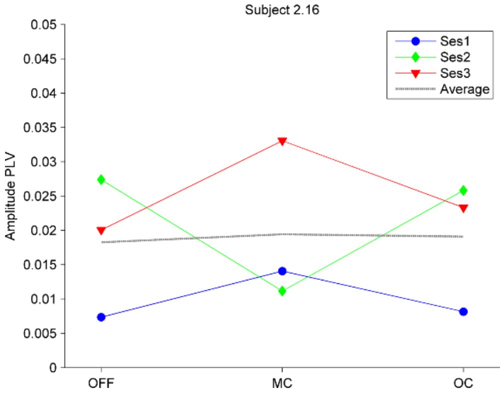
Subject 2.16
Amplitude PLV
Ses1
Ses2
Ses3
Average
OFF
MC
OC

Correlations between Phase Entrainment and Amplitude Modulation Exp1

Scatter plot showing the phase entrainment PLV and amplitude modulation PLV for each of the two session for each subject (same symbols) during each condition in Experiment 1.

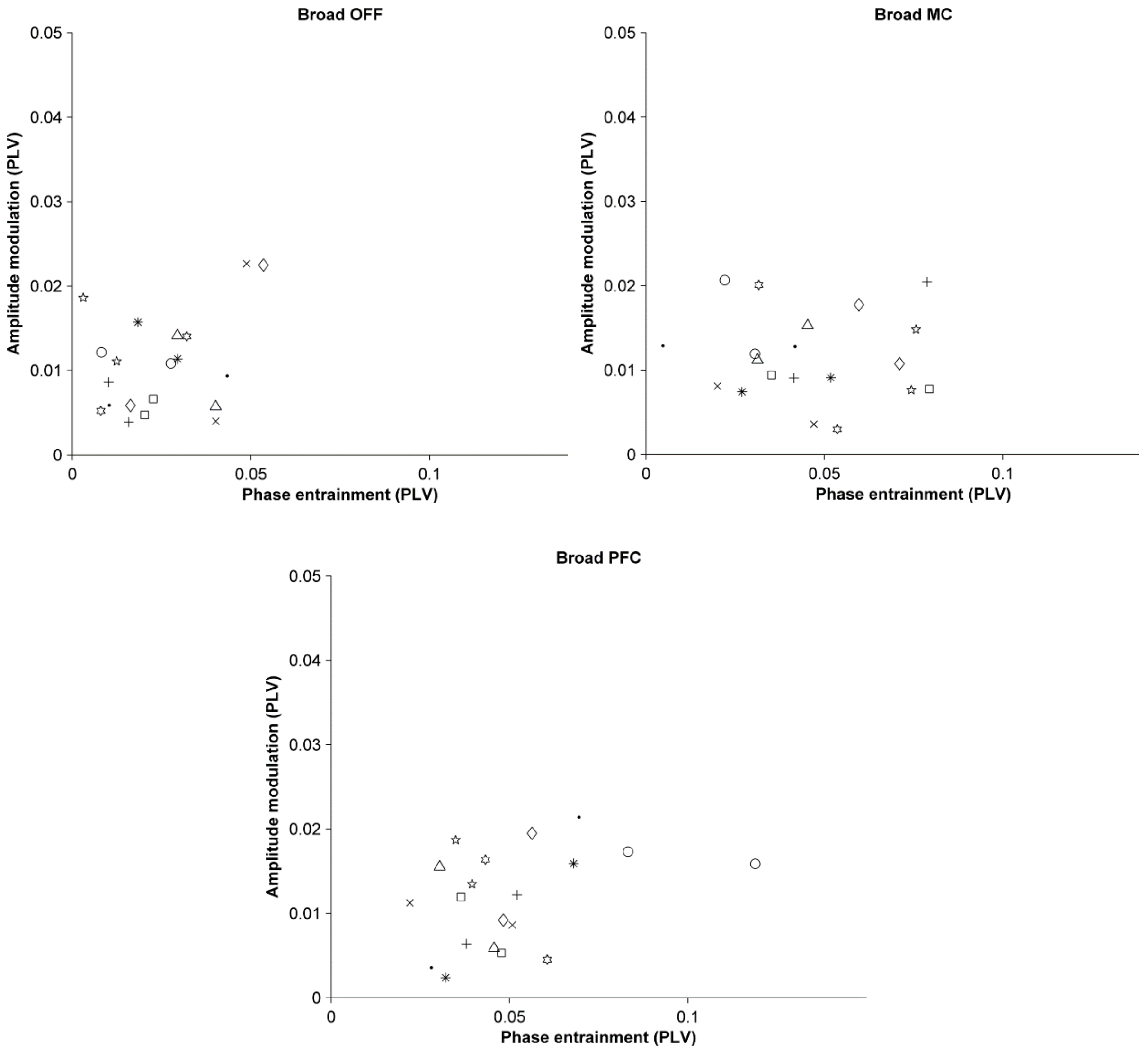

Table 2: The results of the correlation using linear mixed model (Amplitude PLV ~ Phase PLV + (1|Subject)) with the Phase PLV as a fixed effect and Subject as random effect. The P value represents the significance of the fixed effect (Phase PLV) on the Amplitude PLV.

Condition	Estimate	Standard error	P value
OFF	0.13	0.83	0.12
MC	0.01	0.05	0.877
PFC	0.1	0.05	0.077

Correlations Between Phase Entrainment and Amplitude Modulation Exp2

Scatter plot showing the phase entrainment PLV and amplitude modulation PLV for each of the three session for each subject (same symbols) during each condition in Experiment 2.

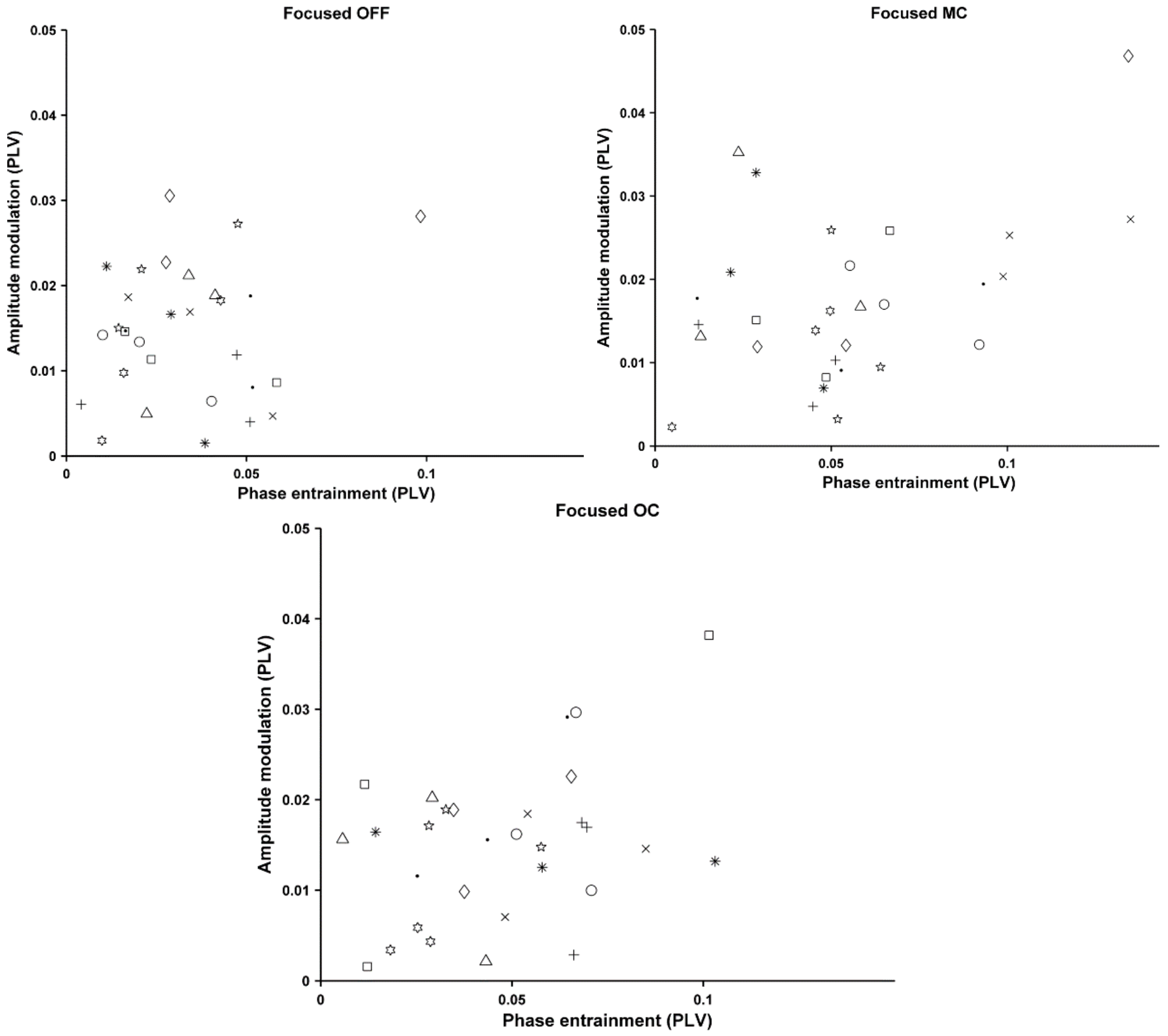

Table 3: The results of the correlation using linear mixed model (Amplitude PLV ~ Phase PLV + (1|Subject)) with the Phase PLV as a fixed effect and Subject as random effect. The P value represents the significance of the fixed effect (Phase PLV) on the Amplitude PLV.

Condition	Estimate	Standard error	P value
OFF	0.03	0.06	0.66
MC	0.12	0.05	0.02
PFC	0.12	0.06	0.037

Correlations Between Phase Entrainment and Amplitude Modulation Exp2-First 2 sessions

Scatter plot showing the phase entrainment PLV and amplitude modulation PLV for the first two sessions for each subject (same symbols) during each condition in Experiment 2.

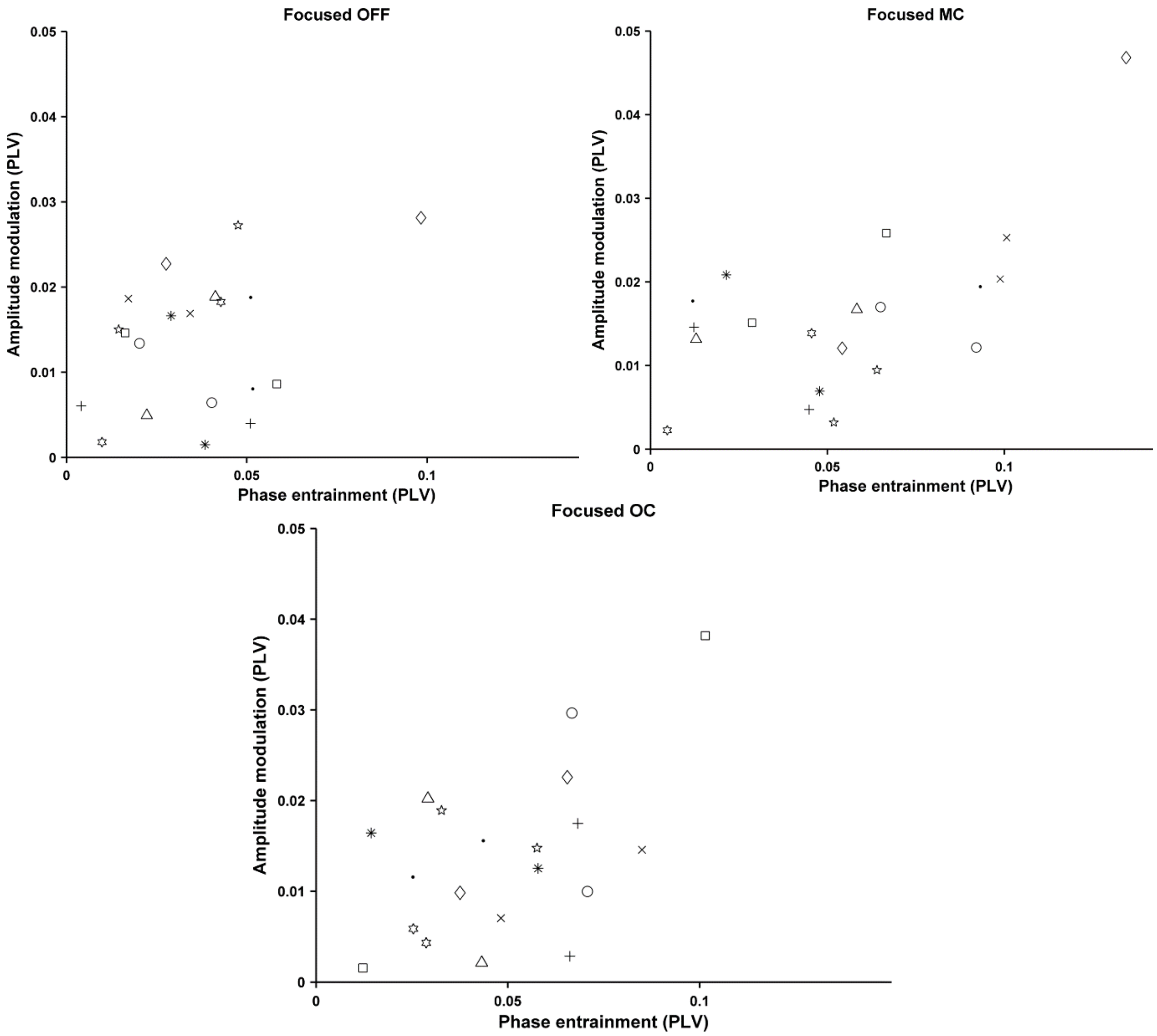

Table 4: The results of the correlation using linear mixed model (Amplitude PLV ~ Phase PLV + (1|Subject)) with the Phase PLV as a fixed effect and Subject as random effect. The P value represents the significance of the fixed effect (Phase PLV) on the Amplitude PLV.

Condition	Estimate	Standard error	P value
OFF	0.14	0.08	0.089
MC	0.17	0.05	0.003
PFC	0.2	0.08	0.013

Data from subject excluded from Exp 2: Difference in tremor frequency and tacs frequency greater than 2 Hz

This subject was unable to maintain a posture with a stable tremor frequency. The subject’s tremor frequency appeared to alternate between either 9.5 or 12.5/13 Hz. In session 1 and 3 tACS was delivered off frequency – at either 9.5 or 12.5 Hz while the dominant tremor frequency was either 13 or 9.5 Hz. PLVs in the MC were low these sessions. In session 2 the highest PLV was observed. Here there appear to be two reasonably equal peaks in the tremor frequency. With the tACS frequency matching the 12.5 Hz. This subject was excluded from Experiment 2.

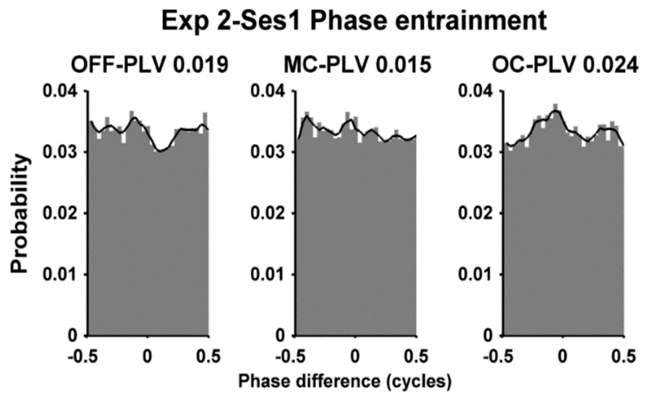

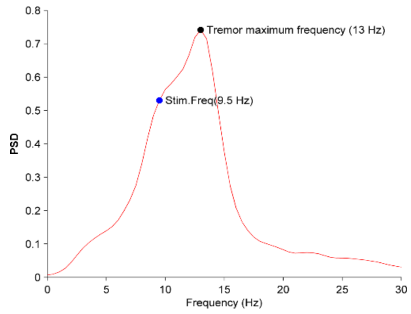

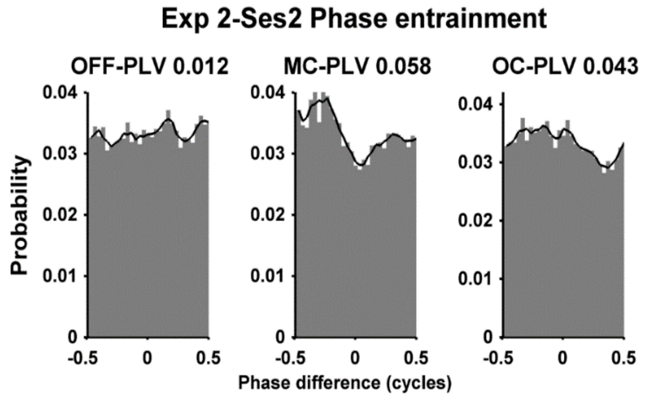

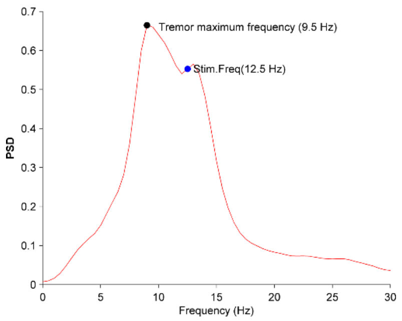

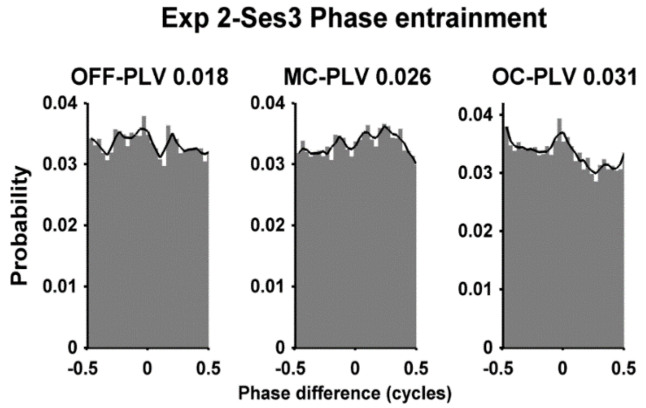

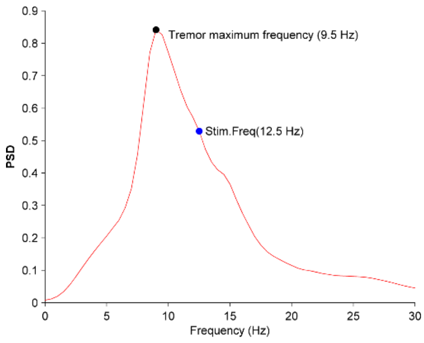

Supplement: Supplementary file 1 — Safety report and supplementary results [file 41598_2018_23290_MOESM1_ESM.pdf]
